# METTL14 promotes intimal hyperplasia through m^6^A-mediated control of vascular smooth muscle dedifferentiation genes

**DOI:** 10.1172/jci.insight.184444

**Published:** 2025-04-17

**Authors:** Grace Chensee, Bob S.L. Lee, Immanuel D. Green, Jessica Tieng, Renhua Song, Natalia Pinello, Quintin Lee, Majid Mehravar, David A. Robinson, Mian Wang, Mary M. Kavurma, Jun Yu, Justin J.L. Wong, Renjing Liu

**Affiliations:** 1 Vascular Epigenetics Laboratory, Victor Chang Cardiac Research Institute, Darlinghurst, New South Wales, Australia.; 2 School of Clinical Medicine, Faculty of Medicine and Health, University of New South Wales, Sydney, New South Wales, Australia.; 3 Epigenetics and RNA Biology Laboratory, School of Medical Sciences, Faculty of Medicine and Health, The University of Sydney, Camperdown, New South Wales, Australia.; 4 Royal Prince Alfred Hospital, Sydney, New South Wales, Australia.; 5 Centre for Peripheral Artery Disease, Heart Research Institute, Newtown, New South Wales, Australia.; 6 Department of Vascular Surgery, National Guangdong Joint Engineering Laboratory for Diagnosis and Treatment of Vascular Diseases, the First Affiliated Hospital for Sun Yat-Sen University, Guangzhou, China.; 7 School of Medical Sciences, Faculty of Medicine and Health, The University of Sydney, Camperdown, New South Wales, Australia.; 8 Center for Metabolic Disease Research and Department of Cardiovascular Sciences, Lewis Katz School of Medicine, Temple University, Philadelphia, Pennsylvania, USA.

**Keywords:** Therapeutics, Vascular biology, Cardiovascular disease, Epigenetics, Gene therapy

## Abstract

Vascular smooth muscle cells (VSMCs) possess significant phenotypic plasticity, shifting between a contractile phenotype and a synthetic state for vascular repair/remodeling. Dysregulated VSMC transformation, marked by excessive proliferation and migration, primarily drives intimal hyperplasia. *N*^6^-methyladenosine (m^6^A), the most prevalent RNA modification in eukaryotes, plays a critical role in gene expression regulation; however, its impact on VSMC plasticity is not fully understood. We investigated the changes in m^6^A modification and its regulatory factors during VSMC phenotypic shifts and their influence on intimal hyperplasia. We demonstrate that METTL14, crucial for m^6^A deposition, significantly promoted VSMC dedifferentiation. METTL14 expression, initially negligible, was elevated in synthetic VSMC cultures, postinjury neointimal VSMCs, and human restenotic arteries. Reducing Mettl14 levels in mouse primary VSMCs decreased prosynthetic genes, suppressing their proliferation and migration. m^6^A-RIP-seq profiling showed key VSMC gene networks undergo altered m^6^A regulation in Mettl14-deficient cells. Mettl14 enhanced Klf4 and Serpine1 expression through increased m^6^A deposition. Local Mettl14 knockdown significantly curbed neointimal formation after arterial injury, and reducing Mettl14 in hyperplastic arteries halted further neointimal development. We show that Mettl14 is a pivotal regulator of VSMC dedifferentiation, influencing Klf4- and Serpine1-mediated phenotypic conversion. Inhibiting METTL14 is a viable strategy for preventing restenosis and halting restenotic occlusions.

## Abstract 2

This study identified inhibition of METTL14 as a critical factor for preventing restenosis and managing restenotic occlusions.

## Graphical abstract

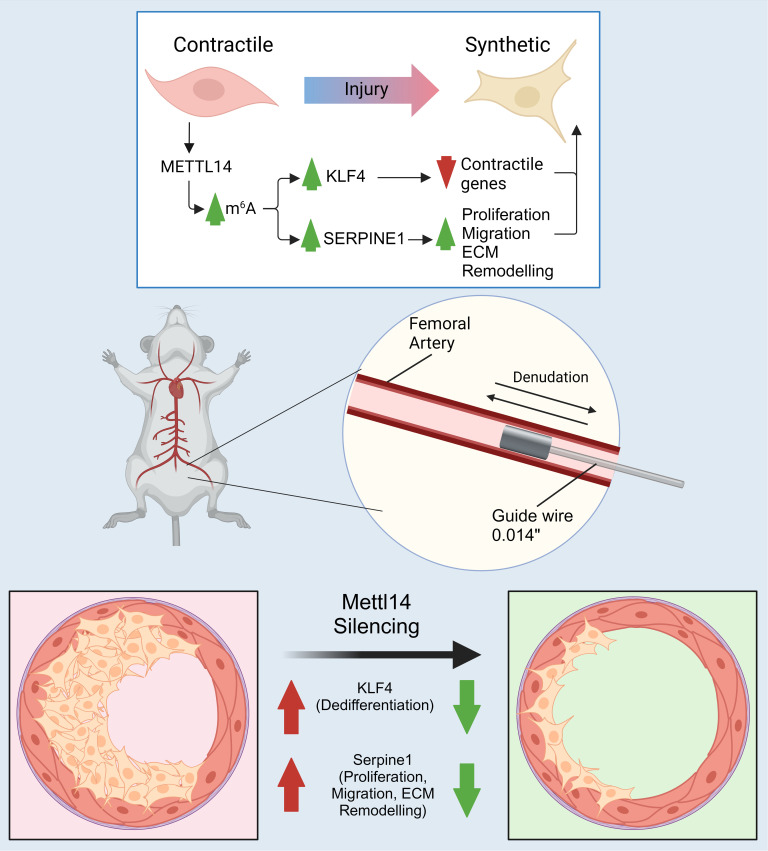

## Introduction

Surgical advancements such as bypass surgery and angioplasty aimed at resuming blood flow to obstructed blood vessels have greatly improved the treatment of stenotic cardiovascular diseases. These interventions are, however, often plagued by intimal hyperplasia, a significant complication characterized by lumen reocclusion that often requires repeated corrective revascularization procedures (1). Phenotypic modulation of vascular smooth muscle cells (VSMCs) serves as the primary substrate for intimal hyperplasia. VSMCs are the predominant constituents of the arterial walls. They are remarkably plastic adult cells that display a physiologically quiescent and contractile phenotype but retain the ability to reenter the cell cycle following injury stimulation (2). This reversibility between phenotypic states allows for normal growth and repair of the vasculature. However, in the context of intimal hyperplasia, the switch to the synthetic phenotype is marked by uncontrolled VSMC proliferation, migration, and secretion of extracellular matrices (ECMs) that result in vessel lumen narrowing and contribute to recurrent stenosis and transplant vasculopathy (3, 4). The inherent plasticity of these cells creates an opportunity for interventions aimed at preventing acute events and/or reversing disease progression.

To develop therapeutic strategies against intimal hyperplasia, the molecular switches that control the VSMC phenotype must be identified and characterized. The expression of VSMC contractile genes is governed by the myocardin (MYOCD) and serum response factor (SRF) complex (5), while Krüppel-like factor 4 (KLF4) is the most well-recognized driver of synthetic VSMCs (6). Histone modification and DNA methylation have been shown to be important players in the activation and repression of these genes (7–9). Recent studies have reported chemical modification on RNA as a new layer of epigenetic regulation, with important roles in biology and diseases (10–12). *N*^6^-methyladenosine (m^6^A) is the most common chemical modification of mRNA in eukaryotes (13), and is implicated in regulation of nearly every aspect of the mRNA life cycle, from mRNA export and stability to translation (14–16). In mammalian cells, the m^6^A modification is catalyzed by the methyltransferase “writer” complex that consists of methyltransferase-like 3 (METTL3) and METTL14 together with auxiliary proteins such as WTAP and VIRMA (13, 17). m^6^A is recognized on mRNAs by “readers” that include the YTH-domain family proteins and can be removed from mRNAs by one of the 2 m^6^A “erasers,” FTO and ALKBH5 (17). Limited data currently exist on how altered expression of m^6^A regulatory proteins affect VSMC phenotype, and even less is known about the role of the m^6^A modification in intimal hyperplasia. Knockdown of Mettl3 or Fto prevented VSMC proliferation and migration in culture (18, 19), while in vivo, METTL3 and WTAP expression was reduced in the neointima of in-stented coronary arteries and rat carotid arteries following balloon injury, respectively (18, 20). Notably, another study also using rat carotid balloon injury showed elevated Mettl3 levels in the neointima (21), whereas loss of Mettl13 in pulmonary arterial smooth muscle cells induced a synthetic cellular phenotype (22). Despite the conflicting findings, these studies collectively suggest an important role for RNA modification in regulating VSMC phenotype and in intimal hyperplasia. Nevertheless, a comprehensive investigation of the transcripts regulated by m^6^A in VSMCs that govern VSMC phenotype and ultimately drive intimal hyperplasia is needed.

Herein, using a combination of cellular assays, animal models, unbiased global m^6^A profiling, and clinical samples, we present the first evidence to our knowledge linking Mettl14 to synthetic VSMCs. We provide clinical validation of METTL14 overexpression in the neointima of human restenotic samples. Knockdown of Mettl14 in synthetic VSMC cultures was sufficient to restore contractile gene expression and normalize proliferation, migration, and ECM production. Using femoral artery wire injury to model intimal hyperplasia, we demonstrate that knockdown of Mettl14 is sufficient to prevent intimal hyperplasia and reduce neointima formation. Mechanistically, we show that crucial synthetic VSMC genes, including *Klf4* and *Serpine1*, are tightly and dynamically regulated by Mettl14-dependent m^6^A modification. Collectively, our findings underscore what we believe is a novel function for Mettl14 in VSMC phenotypic regulation and suggest that targeting the VSMC epitranscriptome via Mettl14 could represent a promising therapeutic strategy for mitigating intimal hyperplasia.

## Results

### METTL14 expression is associated with VSMC phenotypic modulation and intimal thickening.

**Figure 1:**
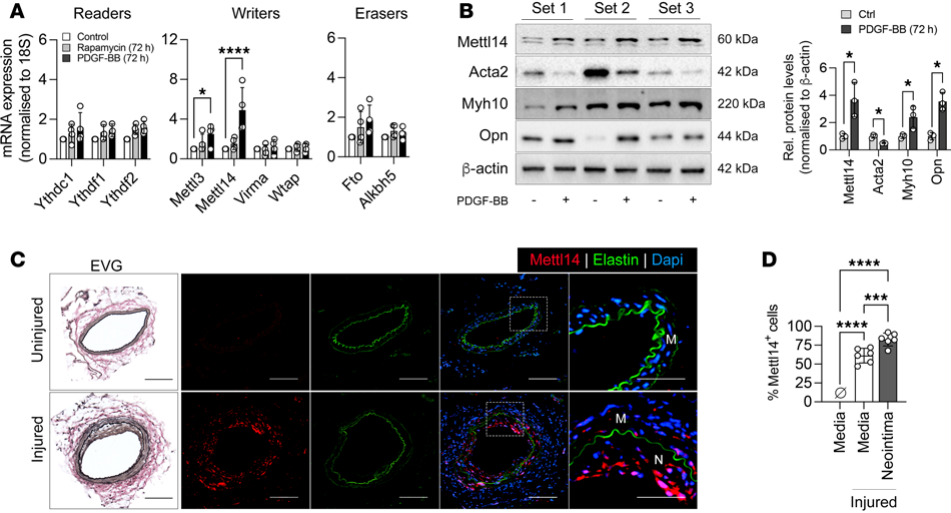
Mettl14 expression is increased in dedifferentiated VSMCs in vitro and following injury. (**A**) mRNA expression of m^6^A modification complex proteins in murine VSMCs treated with rapamycin (50 nM) or PDGF-BB (20 ng/mL) versus control (2% FBS). *n* = 4 independent samples. (**B**) Western blot and quantification of Mettl14 and VSMC markers in murine VSMCs treated without and with PDGF-BB. *n* = 3 biologically independent repeats. (**C**) Representative images of elastin van Gieson (EVG) and immunofluorescently stained cross sections of murine femoral artery 21 days after femoral artery wire injury. Insets were further magnified and presented in the right panels. Mettl14, red; elastin autofluorescence, green; DAPI, blue. M, media; N, neointima. Scale bars: 100 μm. (**D**) Quantification of Mettl14^+^ nuclei in the media of uninjured femoral arteries and in the media and neointima of injured femoral arteries. *n* = 6 biologically independent samples. *P < 0.05; ***P < 0.005; ****P < 0.001 by 2-way ANOVA with Šidák’s multiple-comparison test (**A**), multiple unpaired, 2-tailed Student’s *t* test with Šidák’s multiple-comparison test (**B**), or 1-way ANOVA with Tukey’s multiple-comparison test (**D**). Data are presented as mean ± SD.

To determine the role of mRNA modification in modulating the VSMC phenotype, we induced differentiation and dedifferentiation in murine primary VSMCs using rapamycin and platelet-derived growth factor BB (PDGF-BB), respectively. Gene expression analysis of key m^6^A readers (*Ythdc1*, *Ythdf1*, *Ythdf2*), writers (*Mettl3*, *Mettl14*, *Virma*, *Wtap*), and erasers (*Fto*, *Alkbh5*) revealed a significant upregulation in transcript levels of m^6^A writers *Mettl3* and *Mettl14* in PDGF-BB–treated VSMCs compared with control VSMC cultures (Figure 1A). The gene expression levels of *Virma*, *Wtap*, and m^6^A readers and erasers remained largely unaffected with rapamycin or PDGF-BB treatment. Immunoblotting confirmed the increase in Mettl3 and Mettl14 protein levels following PDGF-BB treatment (Figure 1B and Supplemental Figure 1A; supplemental material available online with this article; https://doi.org/10.1172/jci.insight.184444DS1). This increase coincided with reduced expression of the VSMC contractile marker, α-smooth muscle actin (Acta2), and elevated expression of the synthetic markers non-myosin smooth muscle heavy chain 10 (Myh10) and osteopontin (Opn) (Figure 1B). PDGF-BB is released following vascular injury and is a well-recognized inducer for VSMC proliferation and migration leading to intimal hyperplasia and neointima formation (23). We next induced neointima formation in mouse femoral arteries using a wire injury model (8) to assess the role of Mettl3 and Mettl14 in VSMC phenotypic modulation in vivo. Under physiological/uninjured conditions wherein the majority of VSMCs are in a contractile state, no Mettl3- or Mettl14-expressing cells were detected in the arterial media (Figure 1C and Supplemental Figure 1B). In contrast, the injured media and the neointima formed 21 days after injury was abundant in perinuclear-localized Mettl3^+^ and Mettl14^+^ cells, with the most prevalent and intense staining observed in the neointima (Figure 1C and Supplemental Figure 1B). Over 80% of cells in the neointima expressed Mettl14 compared with approximately 54% of cells being Mettl3^+^ in the injured tissues (Figure 1D and Supplemental Figure 1C). A higher number of Mettl14^+^ cells compared with Mettl3^+^ cells were similarly detected in the injured media (61.22% Mettl14^+^ vs. 35.8% Mettl3^+^ cells; Figure 1D and Supplemental Figure 1C). Considering the more pronounced change was observed with Mettl14 expression, we directed our focus to elucidating its role in VSMC plasticity.

**Figure 2:**
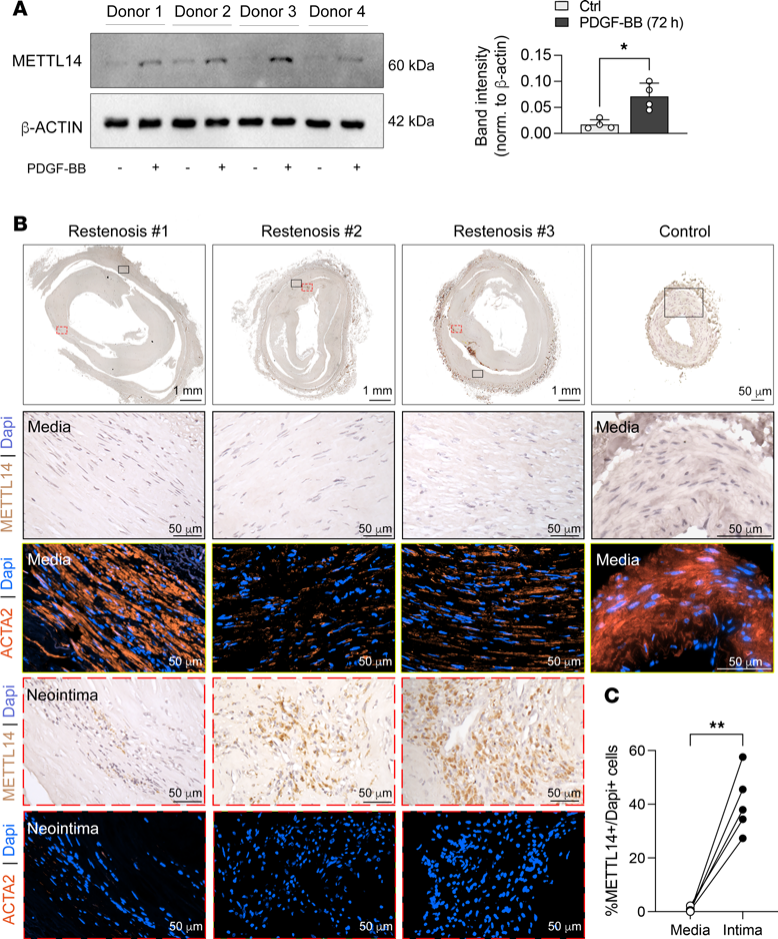
Increased METTL14 expression in the neointima of human restenotic samples. (**A**) Western blot and quantification of METTL14 expression from 4 independent human coronary artery VSMC lines treated with PDGF-BB. (**B**) Representative immunohistochemical staining of METTL14 and immunofluorescent staining of Acta2 in serial sections of femoral arteries from patients. Control arteries were taken from nonrestenotic areas of patients (*n* = 2) and compared to areas with restenosis from patients with arteriosclerosis obliterans (*n* = 5). Top panel: Low magnification of whole femoral artery shown. Middle panel: Magnification of media (black boxed area). Bottom panel: Magnification of neointimal region (red boxed area). METTL14, brown; nuclei, blue; Acta2, red; DAPI, blue. Scale bars: 1 mm (top left 3 images) and 50 μm (all other images). (**C**) Quantification of number of METTL14-expressing cells versus total number of cells from 4 randomly taken images from the media and intimal regions. Each dot represents an individual patient. *P < 0.05; **P < 0.005 by unpaired, 2-tailed Student’s *t* test (**A**) or paired, 2-tailed Student’s *t* test (**C**). Data are presented as mean ± SD.

METTL14 levels were increased in PDGF-BB–stimulated human artery smooth muscle cultures from 4 different donors (Figure 2A). We also determined the expression of METTL14 in human restenotic samples of superficial femoral arteries obtained from patients with chronic limb-threatening ischemia (CLTI). These arteries underwent percutaneous transluminal angioplasty and stenting prior to amputation and artery collection. For normal artery comparison, femoral arteries were collected from areas of amputated legs that did not undergo stenting and with no evidence of restenosis. No METTL14^+^ cells were identified in the media of nonrestenotic control samples (Figure 2B). Low to negligible METTL14 staining was detected in the SMA-rich media of patient tissues (Figure 2B). In agreement with the mouse data, immunohistochemical staining demonstrated upregulation of METTL14^+^ cells in the neointimal regions (Figure 2C). These METTL14^+^ regions did not overlap with SMA expression. Collectively, these in vitro and in vivo findings indicate that METTL14 may be a key regulator in VSMC phenotypic modulation and is associated with the synthetic VSMC phenotype.

### Mettl14 promotes VSMC dedifferentiation.

**Figure 3:**
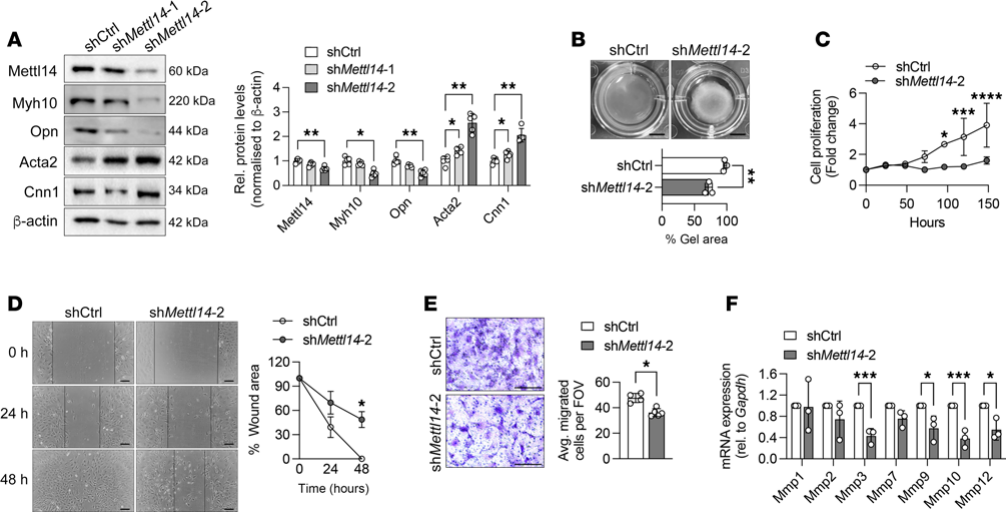
Mett14 silencing promotes VSMC differentiation in vitro. (**A**) Representative Western blot and quantification of Mettl14 expression and VSMC markers in mouse primary VSMCs transduced with lentiviral vectors expressing nontargeting short hairpin RNA (shCtrl) or *Mettl14* shRNA. Two different sh*Mettl14* viruses were tested (sh*Mettl14-1* and sh*Mettl14-2*) and proteins were analyzed 72 hours after transduction. (**B**) Top: Representative image of collagen gel contraction assay of shCtrl- or sh*Mettl14-2*-transduced VSMCs at 72 hours. Bottom: Quantification of gel area. *n* = 3 independent repeats. Scale bars: 5 mm. (**C**) MTS cell proliferation assay of shCtrl- or *shMettl14-2*-transduced VSMCs at 0, 24, 48, 72, 96, 120, and 148 hours. *n* = 4 independent repeats. (**D**) Representative images of scratch assay for cellular migration of shCtrl- or sh*Mettl14-2*-transduced VSMCs at 0, 24, and 48 hours. Lines mark the wound edges of the cultures. Quantification of percentage of wounded area shown on the right. *n* = 4 independent repeats. Scale bars: 50 μm. (**E**) Representative images of Transwell migration assay and quantification of average number of migrated shCtrl- and sh*Mettl14-2*-transduced VSMCs after 48 hours. *n* = 5 independent repeats. Scale bars: 50 μm. (**F**) mRNA expression of matrix metalloproteinases (MMP) in shCtrl- or sh*Mettl14-2*-transduced VSMCs. *n* = 3 independent repeats. *P < 0.05, **P < 0.01, ***P < 0.005, ****P < 0.001 by 2-way ANOVA with Šidák’s multiple-comparison test (**A**, **C**, and **F**), unpaired, 2-tailed Student’s *t* test (**B** and **E**), or repeated measures 2-way ANOVA (**D**). Data are presented as mean ± SD.

To assess the causal role of Mettl14 in VSMC phenotypic modulation, loss-of-function studies were performed using 2 different short hairpin RNAs (shRNAs) to silence *Mettl14* expression in mouse primary VSMCs. Knockdown of Mettl14 reduced the expression levels of Myh10 and Opn and resulted in the upregulation of Acta2 and calponin (Cnn1) (Figure 3A). sh*Mettl14*-2 showed greater knockdown efficiency and was used in all subsequent phenotypic analyses (Figure 3A). Consistent with the increase in contractile protein expression, an approximately 40% increase in VSMC contractility consequent to Mettl14 suppression was observed (Figure 3B). To further examine the impact of reduced *Mettl14* expression levels on VSMC function, we next examined changes in cellular proliferation and migration. VSMCs cultured in normal growth medium and transduced with virus expressing a nontargeting control (shCtrl) showed steady cell growth over time (Figure 3C). Proliferation was minimal in the sh*Mettl14*-2–transduced group cultured under the same medium conditions (Figure 3C). Using scratch wound and Transwell migration assays, knockdown of Mettl14 reduced migratory potential of VSMCs compared with shCtrl cultures (Figure 3, D and E). VSMC migration and proliferation is dependent on the ECM; in particular, the matrix metalloproteinases (MMPs) have established roles in promoting migration and proliferation of VSMCs (24). Quantitative real-time PCR (qRT-PCR) of key MMPs revealed significantly reduced levels of many MMPs in the sh*Mettl14*-2 VSMC cultures compared with the shCtrl cells (Figure 3F). These results further suggest that Mettl14 of the m^6^A writer complex may be involved in promoting a synthetic, injury-responsive phenotype in VSMCs through increased migration, proliferation, and matrix secretion.

### Essential VSMC gene networks and functional pathways are subject to altered m^6^A regulation.

**Figure 4:**
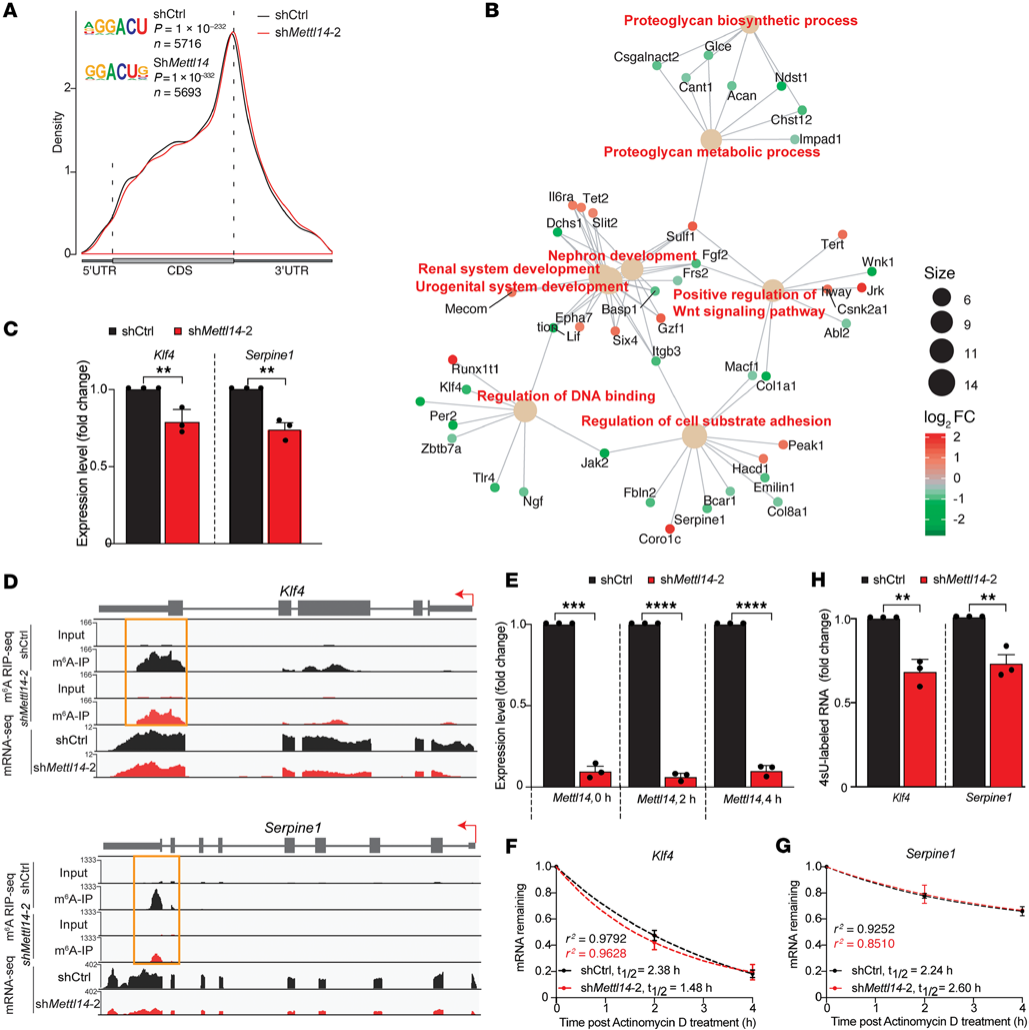
Mettl14 knockdown downregulates the expression of m^6^A-modified mRNAs that control VSMC plasticity. (**A**) Metagene plots showing the distribution of m^6^A peaks detected using m^6^A-RIP-seq and their enrichment within the DRACH motifs in primary mouse VSMCs expressing sh*Mettl14*-2 and shCtrl. (**B**) Cytoscape-generated (https://cytoscape.org/) network map showing m^6^A-modified genes within specific functional pathways that are up- or downregulated in *Mettl14*-knockdown primary mouse VSMCs (sh*Mettl14*-2) compared with control (shCtrl). Up- and downregulated genes are shown in red and green, respectively, and shaded based on the log_2_(fold change) (log_2_FC) values. (**C**) Expression of *Klf4* and *Serpine1* transcripts in primary mouse VSMCs transduced with virus expressing sh*Mettl14*-2 and shCtrl measured using qRT-PCR. (**D**) Integrative Genome Viewer (https://igv.org) plots showing m^6^A peaks on *Klf4* and *Serpine1* mRNAs and the corresponding mRNA expression data in sh*Mettl14-*2–transduced primary mouse VSMCs (red) and shCtrl (black). The m^6^A peaks near stop codons of genes are shown in the orange boxes. (**E**) Levels of *Mettl14* knockdown in cells detected using qRT-PCR at 0, 2, and 4 hours following actinomycin D treatment. (**F** and **G**) RNA stability plots for *Klf4* and *Serpine1* transcripts in *Mettl14*-knockdown VSMCs compared to controls, with decay trend (dotted lines), half-life (*t*_1/2_), and goodness of fit (*r*^2^) shown. (**H**) 4-Thiouridine–labeled (4sU-labeled) nascent RNA transcripts encoded by the *Klf4* and *Serpine1* genes in Mettl14-knockdown VSMCs compared to controls. Data acquired using qRT-PCR were in triplicate from 3 independent experiments. **P *<* 0.01; ***P < 0.001; ****P < 0.0001 by unpaired, 2-tailed Student’s *t* test. Data presented as mean ± SD.

The effects of *Mettl14* deficiency on VSMC phenotypic modulation in vitro and in vivo suggest the possibility of a direct interaction between Mettl14 and key VSMC genes that regulate VSMC dedifferentiation and the injury response. To identify genes that were modified by m^6^A to control VSMC plasticity, we performed and integrated high-throughput mRNA-seq and methylated-RNA immunoprecipitation sequencing (m^6^A-RIP-seq) to determine how m^6^A deficiency affected the VSMC transcriptome in mouse primary VSMCs transduced with virus expressing shCtrl and sh*Mettl14*-2.

m^6^A-RIP-seq revealed that the m^6^A modification was enriched proximal to transcript stop codons (Figure 4A), consistent with previously reported analyses of the m^6^A epitranscriptome (25, 26). Gene ontology (GO) analyses further revealed that m^6^A-modified genes with reduced expression and m^6^A peaks following *Mettl14* depletion were genes involved in ECM production and remodeling (*Col1a1*, *Col18a1*, *Col5a2*, *Fbln2*, *Lpar2*, and *Serpine1*) and injury response such as PDGF-BB, -AA, and -AB signaling (*Pdgfra* and *Pdgfrb*) (Figure 4B, Supplemental Figure 2, and Supplemental Table 1). Most notably, *Klf4* was m^6^A modified and downregulated in *Mettl14*-deficient cells (Figure 4, B and C). Our results indicate m^6^A may be deposited on key VSMC transcripts to control their expression.

We further investigated how Mettl14-mediated m^6^A regulation impacted expression of *Klf4* and *Serpine1* that play major roles in VSMC phenotypic plasticity. KLF4 is a well-recognized factor that drives contractile VSMCs to the synthetic phenotype by repressing contractile gene transcription activation through suppression of MYOCD-SRF complex formation (5, 6, 9). Serpine1 is a serine protease inhibitor found in the ECM and is expressed by VSMCs where it can promote migration and ECM remodeling (27). Each candidate transcript showed a significant (~50%–90%) de-enrichment of m^6^A near the stop codon following *Mettl14* knockdown, concomitant with a decrease in their transcript levels (Figure 4, C and D). Given that m^6^A methylation is known to promote mRNA degradation (15, 28), we performed RNA stability assays following inhibition of transcription using actinomycin D to investigate whether Mettl14 depletion affects the stability of *Klf4* and *Serpine1* transcripts. *Mettl14* deficiency, maintained over the duration of the RNA stability assay (Figure 4E), did not have a notable effect on *Klf4* and *Serpine1* transcript stability (Figure 4, F and G), suggesting that the expression of these transcripts may be regulated through the alternative function of m^6^A as a transcriptional regulator (29, 30). Indeed, we observed a reduction in 4 thiouridine–labeled (4sU-labeled) nascent *Klf4* and *Serpine1* transcripts in *Mettl14*-knockdown VSMCs compared with control cultures (Figure 4H), supporting the hypothesis that the expression of these transcripts is determined by Mettl14-m^6^A–mediated transcriptional control.

### Inhibition of Mettl14 prevents intimal hyperplasia by reducing Klf4 and Serpine1 expression.

**Figure 5:**
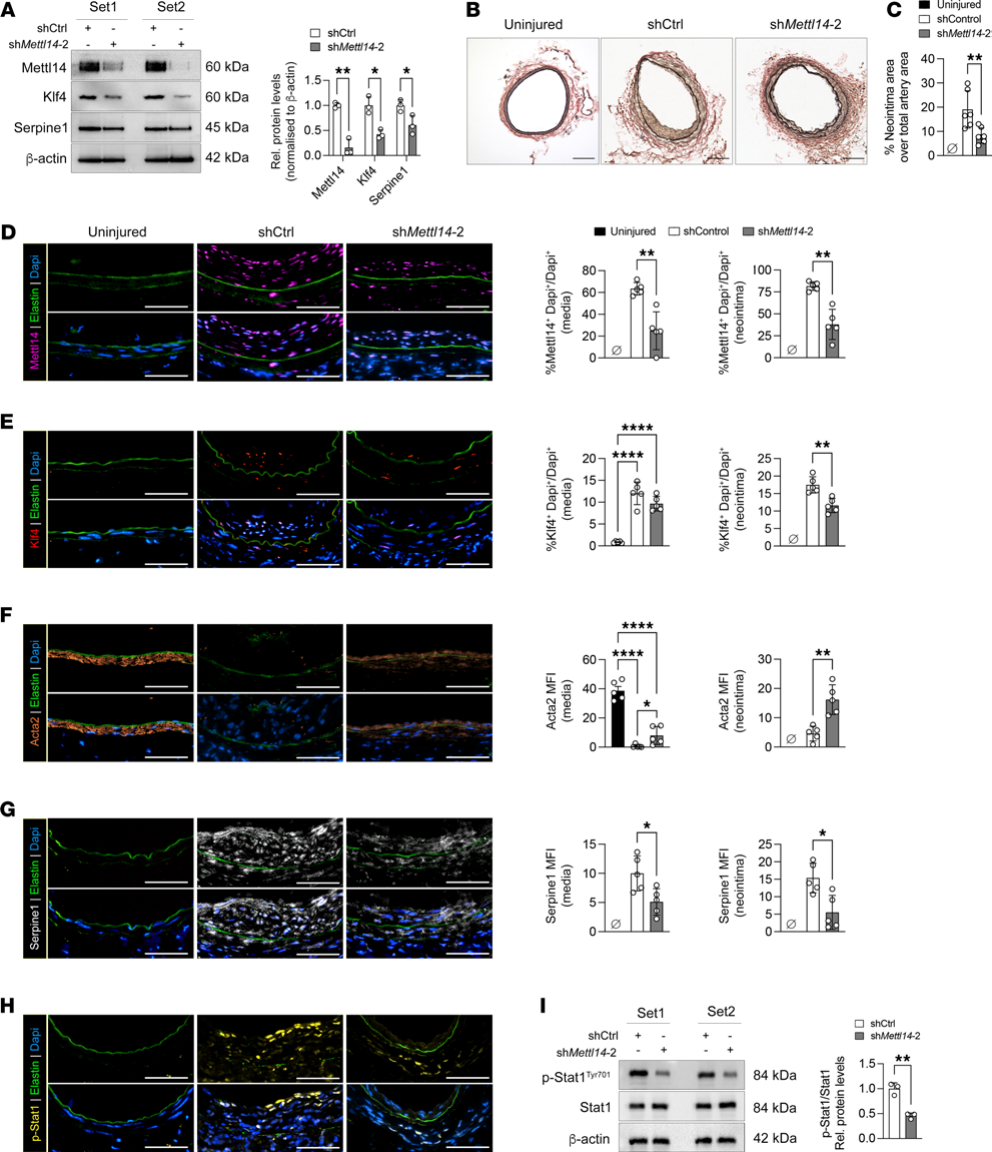
Knockdown of Mettl14 significantly reduced neointima formation in a murine femoral artery wire injury model. (**A**) Representative Western blots and quantification of Mettl14, Klf4, and Serpine1 expression in shCtrl- or sh*Mettl14-2*–transduced VSMCs 48 hours after infection. *n* = 3 independent repeats. (**B**) Representative EVG-stained cross sections of femoral arteries transduced with shCtrl or sh*Mettl14*-2 viruses 3 weeks after wire injury. Scale bars: 100 μm. (**C**) Quantification of neointimal ratio in **B**. *n* = 6 biologically independent samples. (**D**–**G**) Representative Mettl14, Klf4, Acta2, and Serpine1 immunofluorescent staining of murine femoral arteries 21 days after wire injury. Mettl14, purple; Klf4, red; Acta2, orange; Serpine1, white; elastin autofluorescence, green; DAPI, blue. Scale bars: 50 μm. Quantification of cell number or stained area in **D**–**G** is shown on the right. *n* = 5–8 biologically independent samples. MFI, mean fluorescence intensity. (**H**) Representative immunofluorescence images of murine femoral arteries 21 days after wire injury stained for p-Stat1 (Tyr701). p-Stat1, yellow; elastin autofluorescence, green; DAPI, blue. Scale bars: 50 μm. *n* = 5 biologically independent samples. (**I**) Representative Western blot of p-Stat1 and Stat1 expression and their quantification in shCtrl- or sh*Mettl14-2*–transduced VSMCs. *n* = 3 independent repeats. *P < 0.05; **P < 0.01; ****P < 0.001 by multiple unpaired, 2-tailed Student’s *t* test (**A**), 1-way ANOVA with Tukey’s multiple-comparison test (**C** and **D**–**G**), or unpaired, 2-tailed Student’s *t* test (**I**). Data are presented as mean ± SD.

We validated in 2 independent sets of sh*Mettll14*-2 knockdown VSMC cultures that Klf4 and Serpine1 protein levels were also reduced compared with shCtrl cells (Figure 5A), consistent with the decreased *Klf4* and *Serpine1* mRNA expression (Figure 4C). To further assess the role of Mettl14 in VSMC-driven neointima formation, lentiviruses encoding shCtrl or sh*Mettl14*-2 were delivered to femoral arteries of wild-type mice using a pluronic gel viral delivery system during wire injury (8). Analysis of femoral arteries collected 21 days after injury showed that knockdown of Mettl14 markedly inhibited intimal hyperplasia (Figure 5B) and led to significantly reduced neointimal area (Figure 5C). Immunofluorescent staining confirmed that the reduction in neointimal area coincided with efficient knockdown of Mettl14 in both the media and neointima of the sh*Mettl14*-2–transduced injured arteries (Figure 5D). Furthermore, significantly fewer numbers of Klf4-expressing cells were observed in the sh*Mettl14*-2–transduced arteries compared with the shCtrl group (Figure 5E). This loss of Klf4 expression correlated with elevated Acta2 staining in the sh*Mettl14*-2–transduced arteries (Figure 5F), in agreement with our in vitro studies that showed *Mettl14* knockdown could rescue VSMC contractile gene expression (Figure 3A). Moreover, both the neointima and the media of the sh*Mettl14*-2–transduced femoral arteries contained fewer Serpine1^+^ cells compared with arteries transduced with the shCtrl viruses (Figure 5G). Serpine1 can stimulate VSMC proliferation and migration through activation of Janus kinase/signal transducer and activator of transcription (Jak/Stat) signaling (27). In support of this, sh*Mettl14*-2–transduced mice had reduced p-Stat1 (Tyr701) immunostaining, approximately 50% in the injured arteries, a finding also observed in transduced VSMCs (Figure 5I). Collectively, our data suggest that Mettl14, through direct regulation of Klf4 and Serpine1, can impact VSMC phenotype, including contractile gene expression, proliferation, and migration, and extent of intimal hyperplasia.

### Reducing Mettl14 expression in synthetic VSMCs restores characteristics of the differentiated phenotype.

**Figure 6:**
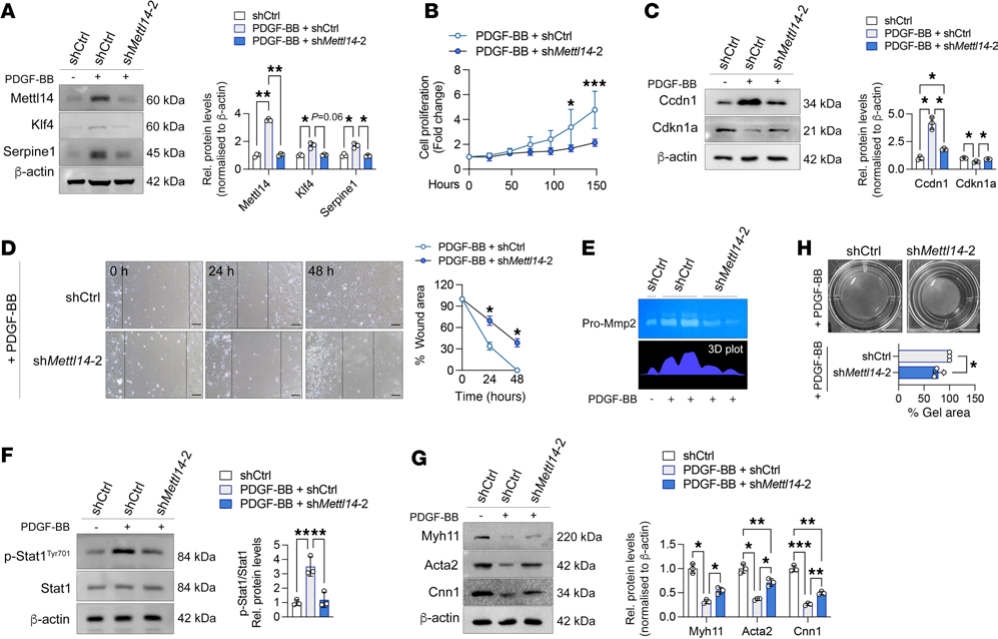
Mettl14 silencing suppresses injury-induced VSMC dedifferentiation in vitro. (**A**) Immunoblotting and quantification of Mettl14, Klf4, and Serpine1 levels in VSMCs pretreated with PDGF-BB or not for 72 hours, followed by transduction with shCtrl or sh*Mettl14-2* viruses. *n* = 4 independent repeats. (**B**) MTS cell proliferation assay performed on murine VSMCs treated with PDGF-BB for 72 hours and then transduced with shCtrl or sh*Mettl14-2* viruses over 148 hours. *n* = 3 independent repeats. (**C**) Representative Western blot and quantification of Ccnd1 and Cdkn1a in shCtrl- and sh*Mettl14*-2–transduced VSMCs following 72 hours of PDGF-BB pretreatment. *n* = 3 independent repeats. (**D**) Representative images of scratch assay assessing cellular migration of PDGF-B–pretreated murine VSMCs transduced with shCtrl or sh*Mettl14-2* at 0, 24, and 48 hours. Lines mark the wound edges of the cultures. Scale bars: 50 μm. Quantification of percentage of wounded area shown on the right. *n* = 3 independent repeats. (**E**) Representative image of gelatin zymography stained with Coomassie blue assessing Mmp2 activity in PDGF-BB–pretreated murine VSMCs transduced with shCtrl or sh*Mettl14-2*. Each lane represents 1 individual sample. *n* = 5 biologically independent samples. Quantification of enzymatic activity in 3D plot shown below. (**F**) Representative Western blot and quantification of p-Stat1 (Tyr701) and Stat1 in PDGF-BB–pretreated murine VSMCs transduced with shCtrl or sh*Mettl14-2*. *n* = 4 independent repeats. (**G**) Representative Western blot and quantification of contractile VSMC markers in PDGF-BB–pretreated murine VSMCs transduced with shCtrl or sh*Mettl14-2*. *n* = 4 independent repeats. (**H**) Representative images of collagen gel contraction assay of PDGF-BB–pretreated murine VSMCs transduced with shCtrl or sh*Mettl14-2*. *n* = 3 independent repeats. Scale bars: 5 mm. *P < 0.05, ***P < 0.005 by 2-way ANOVA with Šidák’s multiple-comparison test (**A**–**C** and **G**), repeated measures 2-way ANOVA (**D**), 1-way ANOVA with Tukey’s multiple-comparison test (**F**), or unpaired, 2-tailed Student’s *t* test (**H**). Data are presented as mean ± SD.

Our data so far demonstrate that the loss of Mettl14 expression can reduce VSMC dedifferentiation (Figure 3) and prevent neointima formation (Figure 5). To enhance the clinical relevance, we aimed to assess whether inhibiting Mettl14 expression in phenotypically modulated VSMCs could restore genetic and functional homeostasis to these synthetic VSMCs, ultimately rescuing intimal hyperplasia in restenotic vessels. To address this, we initially treated VSMCs with PDGF-BB for 72 hours to induce the synthetic state, followed by transduction with either the shCtrl or sh*Mettl14*-2 viruses to determine the effects of Mettl14 loss on VSMC marker expression and functionality. We observed robust knockdown of Mettl14 protein in the PDGF-BB–treated VSMCs that coincided with significant reductions in Klf4 and Serpine1 levels to similar levels as the untreated shCtrl cultures (Figure 6A). Stimulation with PDGF-BB induced high proliferation in the shCtrl VSMC cultures (Figure 6B) consistent with increased cyclin D1 (Ccnd1) and reduced expression of cyclin-dependent kinase inhibitor 1A (Cdkn1a) (Figure 6C). Conversely, knockdown of *Mettl14* in the PDGF-BB–activated cultures almost completely abrogated mitogen-induced cell proliferation while upregulating Cdkn1a expression (Figure 6, B and C). PDGF-BB–induced migration and secretion of Mmp2 were similarly inhibited in the sh*Mettl14*-2–transduced cultures compared with shCtrl-transduced cells (Figure 6, D and E). Treatment with PDGF-BB did affect Stat1 protein expression, but rapidly increased Stat1 phosphorylation at Tyr701, an effect in the sh*Mettl14*-2–transduced cultures (Figure 6F). Importantly, inhibiting Mettl14 in synthetic VSMCs could partially restore VSMC contractile marker expression and rescue contractility in the PDGF-BB–treated, sh*Mettl14*-2–transduced VSMC cultures (Figure 6, G and H).

### Mettl14 inhibition halts intimal hyperplasia in a developing neointima.

**Figure 7:**
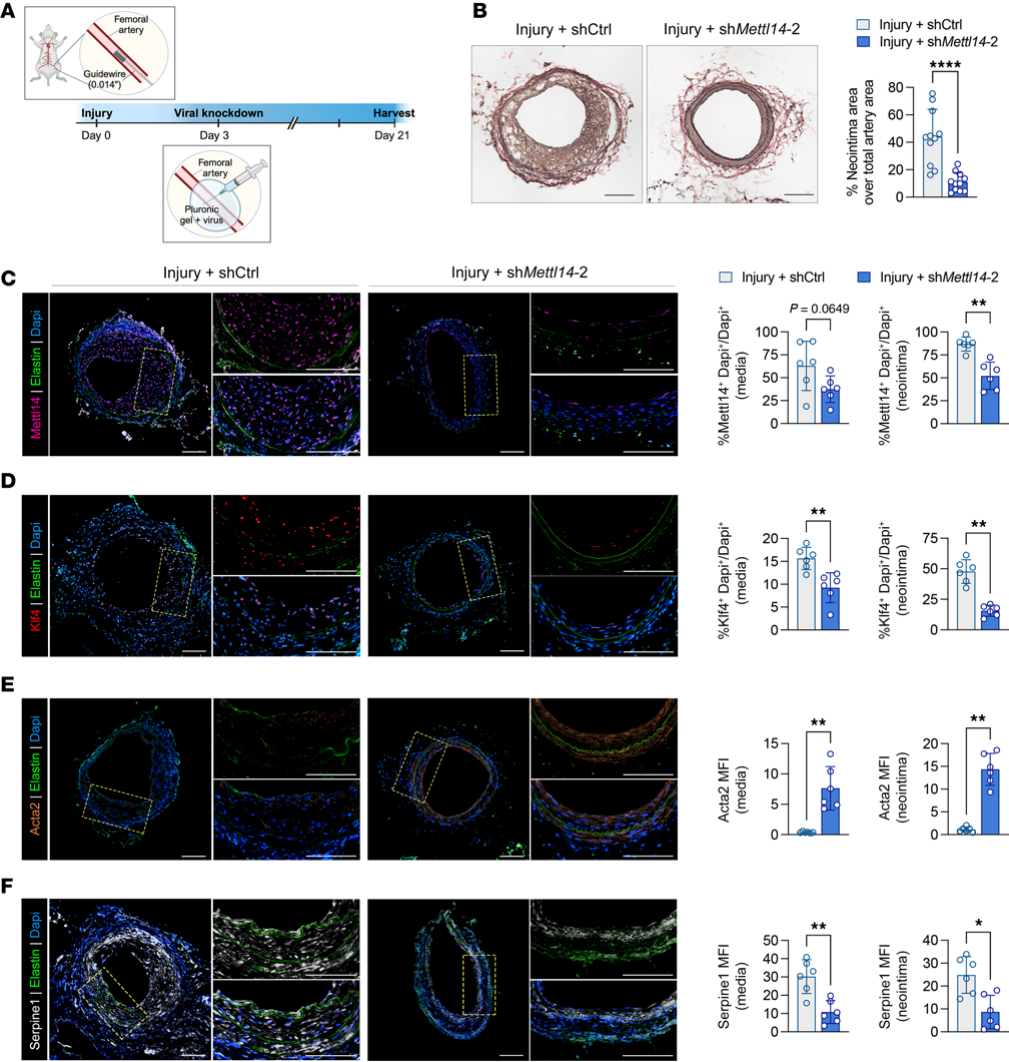
Injury-induced neointima formation can be prevented with early Mettl14 silencing. (**A**) Schematic representation of study design to determine whether METTL14 knockdown in preinjured femoral arteries could attenuate intimal hyperplasia and neointima formation. Viruses were pasted around the injured regions of the femoral arteries 3 days following initial wire injury. Samples were harvested 21 days following injury. (**B**) Representative EVG-stained cross sections of femoral arteries 3 weeks after injury. Quantification of neointimal area shown on the right. Scale bars: 100 μm. (**C**–**F**) Representative immunofluorescence images of murine femoral arteries 21 days after wire injury stained for Mettl14, Klf4, Acta2, and Serpine1. Insets represent zoomed-in neointimal regions that are enlarged in each corresponding right panel. Mettl14, purple; Klf4, red; Acta2, orange; Serpine1, white; elastin autofluorescence, green; DAPI, blue. Scale bars: 100 μm. Quantification of cell number or stained area in **C**–**F** shown on the right. MFI, mean fluorescence intensity. *P < 0.05, **P < 0.01, ****P < 0.001 by 2-way ANOVA with Šidák’s multiple-comparison test. Data are presented as mean ± SD.

**Figure 8:**
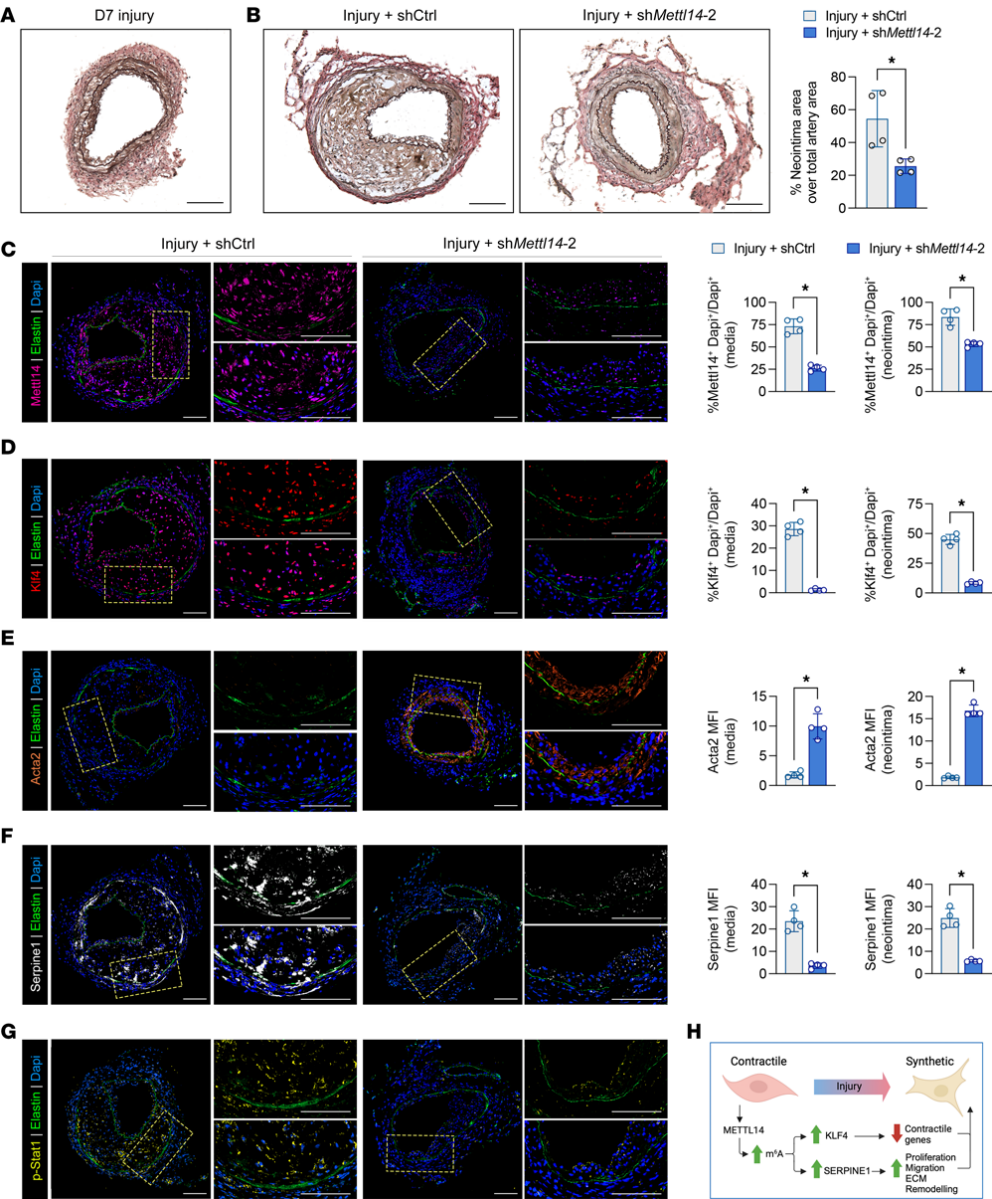
Knockdown of Mettl14 can rescue intimal hyperplasia. (**A**) Representative EVG-stained cross section of day 7 injured femoral arteries. (**B**) Representative EVG-stained cross sections of femoral arteries collected at 3 weeks from arteries treated with shCtrl or sh*Mettl14-2* viruses 7 days after wire injury. Quantification of neointimal area shown on the right. Scale bars: 100 μm. (**C**–**F**) Representative immunofluorescence images of murine femoral arteries 21 days after wire injury stained for Mettl14, Klf4, Acta2, and Serpine1. Insets represent zoomed-in neointimal regions that are enlarged in each corresponding right panel. Mettl14, purple; Klf4, red; Acta2, orange; Serpine1, white; elastin autofluorescence, green; DAPI, blue. Scale bars: 100 μm. Quantification of cell number or stained area in **C**–**F** is shown on the right. (**G**) Representative immunofluorescence images of murine femoral arteries 21 days after wire injury stained for p-Stat1 (Tyr701). Insets represent zoomed-in neointimal regions shown on right. p-Stat1, yellow; elastin autofluorescence, green; DAPI, blue. Scale bars: 100 μm. (**H**) Proposed schematic model of the regulatory effect of Mettl14 on VSMC phenotype. Mettl14 expression is low in abundance in contractile VSMCs and in healthy arteries. Levels of Mettl14 are elevated following injury and lead to increased m^6^A modification of *Klf4* and *Serpine1*, which in turn results in decreased contractile VSMC markers and increased proliferation, migration, and ECM remodeling capabilities As a result, VSMCs change to the synthetic phenotype and participate in intimal hyperplasia. MFI, mean fluorescence intensity. *P < 0.05 by 2-way ANOVA with Šidák’s multiple-comparison test. Data are presented as mean ± SD.

The above in vitro data serve as a proof-of-principle demonstration that by reducing the elevated Mettl14 expression gained during VSMC dedifferentiation, it is possible to restore the contractility, proliferation, and migratory capabilities of synthetic VSMCs to that of a more differentiated cell type. To determine whether redifferentiation of synthetic VSMCs was also possible in vivo, we repeated the femoral artery wire injury experiments in wild-type mice, followed 3 days later by viral knockdown of Mettl14 3, a time when VSMCs are in a state that is primed for proliferation and migration to initiate neointima formation (31, 32) (Figure 7A). Delivery of the sh*Mettl14*-2 virus subsequent to femoral wire injury effectively suppressed the formation of neointima compared with the shCtrl-transduced injured group (Figure 7B). Immunofluorescent staining confirmed significant knockdown of Mettl14 in synthetic VSMCs in vivo (Figure 7C), where this loss of Mettl14 was particularly evident in the neointimal regions. Reduction of Mettl14 in injured arteries resulted in strong reductions in Klf4 and Serpine1 levels in both the media and neointima (Figure 7, C–E). We demonstrate that these reductions in Klf4 and Serpine1 led to upregulation of Acta2 (Figure 7F). To further investigate whether Mettl14 knockdown could rescue an artery with more advanced neointima formation, we delivered sh*Mettl14*-2 viruses to femoral arteries 7 days after injury. A thin layer of neointima was already evident at this time point (Figure 8A). Similar to day 3 intervention, knockdown of *Mettl14* in arteries 7 days after injury significantly attenuated neointima formation (Figure 8, B and C). Similarly, we observed a reduction in Klf4 and Serpine1 levels that coincided with an increase in Acta2 expression (Figure 8, D–F). Furthermore, p-Stat1 expression was also reduced (Figure 8G). We propose a model wherein Mettl14 inhibition simultaneously promotes VSMC contractile gene expression via reduced Klf4 activation while limiting VSMC proliferation and migration through reduced Serpine1 and Jak/Stat1 signaling (Figure 8H).

## Discussion

METTL14 has a reported role in the pathobiology of many diseases (10–12, 33–35). Until now, the function of METTL14 in VSMCs has remained largely unknown. In the current study, we identified METTL14 as a critical RNA-modifying protein that drives VSMC dedifferentiation by facilitating the deposition of m^6^A on key VSMC transcription factor mRNAs, including the master driver of synthetic VSMCs, *Klf4*. Of clinical relevance, we observed upregulation of METTL14 in the neointima of clinical restenosis samples, a region that is densely populated by phenotypically modulated VSMCs. Through a series of in vitro and in vivo experiments, we confirmed that Mettl14 directly regulates VSMC phenotypic switching and silencing of Mettl14 can limit intimal hyperplasia, offering it as a potential preventive and interventional therapeutic option.

Among the mRNA readers, writers, and erasers, our study specifically highlighted an increase in Mettl3 and Mettl14 expression during VSMC phenotypic modulation in vitro as well as elevated expression in neointimal regions in mouse and human tissues. These results align with previous research demonstrating upregulation of Mettl3 expression in phenotypically modulated VSMCs in culture and in the neointima of rat carotid arteries following balloon injury (21). However, in contrast with our observations, Zhao and colleagues observed no significant changes in Mettl14 expression. This difference could be attributed to the different starting materials used to screen for m^6^A modifiers. For example, our study identified upregulation of Mettl3 and Mettl14 in a pure VSMC culture environment following PDGF-BB–induced dedifferentiation, whereas Zhao and coworkers performed mRNA analysis on injured rat carotid arteries where various other cell types such as endothelial cells and inflammatory cells are also present and could mask expression in VSMCs. Indeed, elevated Mettl3 expression in endothelial cells (36, 37) and peritoneal macrophages (38) has been previously reported. The heightened expression in non–VSMC-lineage cells may be accounted for predominantly by medial localization of Mettl13 in the balloon-injured rat carotid arteries and limited expression detected in the neointima in that study (21). Nevertheless, both our study and Zhao’s underscore the important role of the m^6^A writers in VSMC phenotypic regulation. In further support of our data, others have reported high METTL14 expression in human abdominal aortic aneurysms and dissections (39) and vascular calcification (40), pathologies associated with significant VSMC dedifferentiation. Although we cannot attribute the effect of intimal hyperplasia to Mettl14 alone, changes in Mettl14 expression represent a critical determinant in VSMC phenotypic modulation and neointima formation.

The identification of *Klf4* and *Serpine1* as downstream m^6^A-modified targets of Mettl14 in VSMCs indicates the critical role of Mettl14 in regulating the expression of these key VSMC genes (Figure 4). In agreement, a recent study has shown Klf4 to be a target of Mettl3 in pulmonary arterial smooth muscle cells (22), while regulation of Serpine1 via RNA m^6^A methylation in VSMCs has not yet been reported elsewhere. While we show that Mettl14 depletion downregulates nascent transcript expression of these genes, further studies are required to determine whether this effect is m^6^A dependent, given that other studies have reported Mettl14 can regulate transcription independently of m^6^A (41). Our study further identified a reduction in Jak/Stat signaling in response to the loss of Mettl14. Activation of Jak/Stat signaling promotes VSMC proliferation, migration, and dedifferentiation (27, 42). We propose a model where we place Mettl14 and its associated m^6^A modification at the center of a signaling network that involves Klf4, Serpine1, and Jak/Stat activation to govern VSMC phenotype and intimal hyperplasia (Figure 8H). m^6^A methylation may also regulate the expression and functions of other genes that are involved in Jak signaling and ECM remodeling, including various collagens such as collagen 1 and fibulin (Figure 4B). Further functional assays are warranted to define how m^6^A regulates these genes during VSMC phenotypic switching.

A notable limitation of most current research in intimal hyperplasia lies in the predominant use of prevention models, wherein genes are typically knocked down in healthy arteries to elucidate their impact on disease development. In the clinic, however, interventional procedures are performed on patients already presenting with vascular complications. To address this disparity and further test the clinical relevance of our work, we performed an additional interventional approach that aimed to determine whether inhibiting Mettl14 expression could reduce neointima formation in arteries undergoing intimal hyperplasia. We targeted both an early stage after injury (3 days) when VSMCs are priming for intimal hyperplasia (31, 32), as well as 7 days after injury when a layer of neointimal lesions within the internal elastic lamina is present (43, 44). Blocking Mettl14 at these critical junctures could halt neointima formation by partially restoring contractile gene expression and contractile function in VSMCs, while simultaneously limiting their proliferation and migration potentials (Figure 6 and 7). It would be of interest to determine whether Mettl14 inhibition could lead to regression of a neointima in an artery with advanced restenosis (i.e., at 3 weeks after injury). The difficulty with this approach lies in identifying the diseased arteries and following changes in neointima size in live mice with interventional therapies. Current imaging modalities to image femoral arteries are limited in resolution, and while optical coherence tomography has been used to assess flow in live mouse femoral arteries (45), its ability to accurately measure neointimal area has not been shown. Nevertheless, our data show that incorporating a strategy to include METTL14 inhibitors into drug-eluting stents may be an effective strategy to treat restenosis, reducing and/or eliminating the need for future corrective procedures. Supported by our mechanistic data, inhibiting METTL14 may represent a superior strategy compared with paclitaxel and rapamycin that are currently used to target VSMC proliferation since METTL14 can regulate all aspects of VSMC dedifferentiation (contractile, migration, and proliferation). Notably, excessive VSMC proliferation and migration play pivotal roles in the development of various vascular occlusive diseases, including atherosclerosis, and contribute to heart transplant and vein bypass graft failures. The effectiveness of inhibiting METTL14 in reducing the disease phenotype under these conditions warrants further investigations. Furthermore, as vascular diseases are not caused by one cell type but involve an interplay between multiple cellular populations, including inflammatory cells and the endothelium, and with raising awareness of the role of adventitial cells, it would be of interest to determine whether our interventional study in tissue-specific Mettl14-knockout mice would generate similar therapeutic benefits.

Despite METTL14 having no inherent catalytic activity during m^6^A modification, METTL14 is essential for recognizing RNA substrates. Studies to date have shown that METTL14 can be preferentially reduced, relative to METTL3, in a variety of cellular contexts and have profound effects on cell development, cancer, and late-stage heart failure (10–12, 33–35). As METTL14 stabilizes METTL3 to deposit m^6^A, a decrease in METTL14 expression alone may be sufficient to lower overall m^6^A levels and affect cellular phenotype and signaling. Our study adds another dimension to the developing landscape of epigenetic regulation of gene expression in VSMC phenotypic modulation and in vascular injury. Our findings open intriguing possibilities for controlling VSMC phenotype and pathological remodeling of arteries through targeting METTL14.

## Methods

### Sex as a biological variable.

Both female and male mice were used throughout this study. Findings were similar for both sexes and data are presented together. Human samples used were also from male and female patients.

### Human samples.

Arterial samples were obtained from amputated legs of patients who were diagnosed with arteriosclerosis obliterans based on echo and/or CT and suffered from CLTI. Prior to amputation, the limbs underwent percutaneous transluminal angioplasty and stenting. The superficial femoral arteries were collected from the amputated limbs and processed for paraffin embedding using standard procedures. Control nonrestenotic arteries were collected from patients with CLTI undergoing below-knee amputation at the Royal Prince Alfred Hospital. Arteries (~100–200 μm in diameter) from subcutaneous fat surrounding the tibialis anterior were collected and fixed in formalin.

### Femoral artery wire injury.

Eight- to 10-week-old C57BL/6J mice (Australian Bio Resources) underwent femoral artery wire injury as a model of postangioplasty restenosis to study intimal hyperplasia, as previously described (8). All animals were first anesthetized with 2% isoflurane at 90% O^2^. Upon loss of pedal reflex, a 0.014-inch guidewire was inserted into the femoral artery and left for 45 seconds to overstretch the arterial wall. For gene knockdown experiments, injury was performed as above and viruses (1 × 10^7^ PFU) were delivered by painting the concentrated viral supernatant and pluronic-127 gel (Sigma-Aldrich) mixture circumferentially around the injured femoral artery. The contralateral uninjured femoral artery was used as the baseline control. Femoral arteries were harvested 3 weeks after surgery and analyzed in a blinded manner. Animals were euthanized by 3% isoflurane with cardiac puncture perfusion of PBS, and a final cervical dislocation prior to tissue collection.

### Immunostaining, microscopy, and quantification.

Femoral arteries were harvested and fixed in 4% paraformaldehyde overnight at 4°C followed by dehydration in 30% sucrose overnight. Samples were embedded in OCT (Tissue-Tek, Sakura) and sectioned (Leica Biosystems). Hematoxylin-eosin (H&E) staining was performed using standard protocols. Elastin van Gieson (EVG) staining was performed following the manufacturer’s protocol (Australian Biostains). For immunofluorescent staining, cryosections were blocked with 5% BSA for 1 hour and sections were incubated with primary antibodies at 4°C overnight. Following incubation with secondary antibodies, images were mounted with DAPI-mount (Thermo Fisher Scientific). Sections stained with secondary antibodies alone were used as controls (Supplemental Figure 3). Bright-field images were acquired with the Leica DM4000 B LED microscope and fluorescence images captured using a Nikon Eclipse Ti-2E. A mean value was generated from a minimum of 3 independent sections of each artery sample. The mean fluorescence intensity (MFI) of SERPINE1 and ACTA2 were quantified in the regions of interest using ImageJ software (NIH) as previously described (47). Cell number and intimal and medial area quantifications were analyzed using ImageJ. Antibodies used in the study are listed in Supplemental Table 2.

### Cell culture.

HEK293T cells (ATCC) were grown in DMEM (Gibco) supplemented with 10% FBS (Hyclone) and 1% penicillin-streptomycin (Gibco). Mouse primary VSMCs were isolated from the descending aorta of 8- to 12-week-old C57BL/6J mice as previously described (48). Briefly, the descending aortas were incubated in an enzymatic cocktail containing 1.25 U/mL elastase (Sigma-Aldrich) and 175 U/mL collagenase (Sigma-Aldrich) for 10–15 minutes at 37°C with shaking to remove the adventitia. The stripped aortas were then cut longitudinally and the endothelium scraped. Aortas were then plated onto tissue culture plates and VSMCs allowed to migrate out. Cells were cultured in DMEM/F-12 (Gibco) supplemented with 15% FBS and 1% penicillin-streptomycin. Human coronary artery smooth muscle cells were purchased from Lonza and cultured as previously described (48). Murine and human VSMCs were used between passage 5 and 7. To induce phenotypic switching of VSMCs, cells were fasted in 2% FBS starvation media overnight prior to treatment with rapamycin (50 nM; PeproTech) or PDGF-BB (20 ng/mL; PeproTech) to induce differentiation and dedifferentiation, respectively (8, 48).

### VSMC proliferation, migration, and contraction assays.

Cell proliferation was determined using the CellTiter 96 Aqueous One MTS assay according to manufacturer’s instructions (Promega). In brief, dye solution was added to 96-well plates seeded with 0.4 × 10^4^ cells per well. Following a 4-hour incubation period, solubilization solution was added to each well and absorbance read at 570 nm (PheraStar FS). VSMC migration was assessed using a wound healing scratch assay where a scratch was made across the well of fully confluent VSMCs; cell migration into the denuded zone was observed over 48 hours and percentage of gap closure was calculated using ImageJ. Transwell migration assays were also used to further assess VSMC migration. Eight-micrometer-pore membrane inserts (Corning) were coated with 0.1% gelatin and VSMCs (4 × 10^4^ cells) were loaded into the upper chamber in 0.1% FBS–containing medium and then exposed to 10% FBS–containing medium in the lower chamber for 48 hours. Membranes were fixed with 4% paraformaldehyde for 20 minutes and stained with 0.1% crystal violet in the dark for 15 minutes. Images of the membrane underside and migrated cells were captured using a Zeiss microscope and quantified by ImageJ in a blinded manner. VSMC contraction was determined using a cell contraction assay kit (CytoSelect) per the manufacturer’s instructions. Collagen gel size change (contraction index) was monitored over 48 hours and measured using ImageJ.

### Gelatin zymography.

Gelatinase substrate gel electrophoresis was performed using 10% polyacrylamide gels with 0.1% gelatin. Samples were prepared in loading buffer consisting of 0.4 mol/L Tris, pH 6.8, 5% SDS, and 0.03% bromophenol blue. Gels were washed in renature solution (2.5% Triton X-100) for 1 hour at room temperature and then incubated in substrate buffer (50 mmol/L Tris, pH 7.5, 10 mmol/L CaCl_2_, and 1 mmol/L ZnSO_4_) at 37°C overnight. Gels were stained with Coomassie blue to reveal regions of gelatinase activity. The 3D plot was generated with ImageJ software using the Interactive 3D surface plot plugin.

### shRNA lentiviral vector production and transduction.

Viral supernatants were produced by calcium phosphate transfection of HEK293T cells (3–4 × 10^6^ cells/well) with pRSV-Rev, pMDLg/p.rre, and pMD2.VSV-G used to package lentiviruses. Media were replaced with fresh 10 mL HEK293T growth medium supplemented with 25 μM chloroquine 1 hour before transfection. Cells were given fresh HEK293T medium supplemented with 2 mM sodium butyrate 24 hours after transfection. Lentivirus-containing supernatants were harvested 24 hours later via centrifugation at 350*g* for 5 minutes at room temperature to pellet the cells. Supernatant was removed and filtered using a 0.45 μm filter. Filtered lentivirus-containing supernatants were aliquoted into 1.5 mL Eppendorf tubes and snap-frozen on dry ice and stored at –80°C for later use. For in vivo use, filtered supernatant was concentrated by ultracentrifugation for 2 hours at 20,000 rpm in a Beckman SW28 rotor. Viral supernatant was resuspended on ice in DMEM at 1/200^th^ of the original volume.

VSMCs were seeded into 6-well plates to reach a confluence of 70% before transduction. Cells were incubated for 1 hour in DMEM containing 15% FBS, 1% penicillin-streptomycin, and 4 μg/mL polybrene. Cells were infected with lentiviral supernatant for 24 hours. The nontargeting control lentiviral vector used was ath-miR-159 pLKO.1-2A-GFP that expresses an shRNA against a sequence encoding plant microRNA. Fresh medium was added to transduced VSMC cultures and cells were collected 48 hours later. Success of lentiviral transduction was verified by visualizing eGFP expression with a Nikon Eclipse Ti-E inverted microscope. Knockdown efficiency was confirmed by Western blotting and/or qRT-PCR.

### RNA purification and qRT-PCR.

Total RNA was isolated using TRIzol reagent (Invitrogen) and reverse transcribed using an iScript cDNA synthesis kit (Bio-Rad) according to the manufacturers’ instructions. qRT-PCR was performed on diluted cDNA with SsoFast EvaGreen (Bio-Rad) and on a Bio-Rad Real-Time PCR machine. Analysis was performed using the accompanying CFX Manager analysis software. *18S* was used as the housekeeping control. Primers are provided in Supplemental Table 3.

### Western blot.

Cells were lysed in radioimmunoprecipitation assay (RIPA) buffer (Thermo Fisher Scientific) supplemented with 1% (v/v) Halt PIC (Thermo Fisher Scientific). The protein concentration was determined using a BCA protein assay kit (Thermo Fisher Scientific) followed by standard Western blot protocols. Membranes were incubated with respective primary antibodies overnight at 4°C and then incubated with corresponding secondary antibodies for 1 hour at room temperature. The reaction was followed by ECL+ detection system (Thermo Fisher Scientific) and then exposed using a Bio-Rad ChemiDoc Touch Imaging System. The results were expressed as fold induction by normalizing the data to control values. A list of antibodies used is provided in Supplemental Table 4. Blots per figure panel were run in parallel and presented together with their respective loading control.

### RNA stability assay.

Control and *Mettl14*-depleted VSMCs were treated with 10 μg/mL actinomycin D (Sigma-Aldrich) for different time points (e.g., 0, 2, and 4 hours) and harvested. RNA extraction was performed on harvested cells using TRIzol reagent. cDNA synthesis and qRT-PCR were performed as described above. RNA decay rates were determined using nonlinear regression curve fitting (1-phase decay), with the following parameters: least squares (ordinary fit), confidence level 95% and asymmetrical (likelihood) CI, with medium convergence criteria, as previously described (49).

### mRNA- and m^6^A-RIP-seq.

To determine gene expression changes between shCtrl- and sh*Mettl14*-transduced primary murine VSMCs, poly-A–enriched RNA samples, each in duplicate, were prepared and sent for mRNA-seq analysis. Briefly, poly-A–enriched mRNA libraries were prepared from 2 μg of TURBO DNase–treated, total RNA using the TruSeq Stranded Library Preparation Kit (Illumina), according to the manufacturer’s instructions. mRNA-seq was performed on each cell population in duplicate by Novogene. A total of 100 million paired-end strand-specific reads were sequenced per sample on an Illumina HiSeq 2500 platform.

To determine accompanying m^6^A epitranscriptomic changes, samples were prepared for m^6^A-RIP-seq as previously described (26). Briefly, 3–5 μg total RNA underwent zinc-mediated fragmentation into approximately 200-nt-long fragments, verified by using a Bioanalyzer (Agilent Technologies). To enrich for RNA fragments containing m^6^A modifications, magnetic beads were first prepared in IP buffer and 5 μg anti-m^6^A antibody (Millipore, ABE572) by mixing for 6 hours at 4°C. The antibody-bead mixtures were resuspended in IP reaction solutions containing fragmented total RNA. m^6^A-enriched fragmented RNA was eluted from the beads using the RNeasy Mini Kit (Qiagen). The eluates were resuspended in ethanol and transferred to an RNeasy MiniElute spin column (Qiagen) and centrifuged at high speed. m^6^A-enriched RNA fragments, resuspended in nuclease-free water, then underwent library preparation and m^6^A-RIP-seq at a depth of 20 million single-end reads, per sample, using an Illumina NextSeq 500 high-output platform, as previously described (26).

### Nascent RNA assay.

Nascent RNA was labeled with 4sU and captured following the method described by Lee et al. (50). Briefly, 1 × 10^7^ VSMCs were cultured in a 10-cm plate with growth media and transduced with either a sh*Mettl14*-2 or nontargeting control shRNA. Seventy-two hours after transduction, the media were replaced with fresh media containing 700 μM 4sU for up to 15 minutes. Cells were lysed by adding 3 mL TRIzol directly to each plate. Total RNA was extracted using according to the manufacturer’s instructions and diluted in 120 μL nuclease-free water. Five percent of the total RNA was reserved for validating the *Mettl14* knockdown levels.

The remaining RNA was biotinylated in a 250 μL reaction comprising 50 μL MTSEA biotin-XX (166 mg/mL in DMF; Biotium Inc), 25 μL 10× biotinylation buffer (100 mM Tris, pH 7.4; 10 mM EDTA), 115 μL RNA, and 60 μL water. The biotinylation reaction was incubated at room temperature in the dark for 2 hours on a rotator. RNA was then extracted using acidic phenol (pH 4.5) following standard protocols. The purified RNA was resuspended in 250 μL nuclease-free water, diluted by 2-fold in 2× high-salt buffer (100 mM Tris-HCl pH 7.4, 2 M NaCl, 20 mM EDTA, 0.1% Tween 20), and incubated with 20 μL Dynabeads MyOne Streptavidin C1 beads (Invitrogen) at room temperature for 30 minutes on a rotator. The beads were washed 3 times with high-salt buffer (50 mM Tris-HCl pH 7.4, 1 M NaCl, 10 mM EDTA, 0.05% Tween 20), twice with TET buffer (10 mM Tris-HCl pH 7.4, 1 mM EDTA, 0.05% Tween 20), and once with TE buffer (10 mM Tris-HCl pH 7.4, 1 mM EDTA). To elute the 4sU-labeled nascent RNA, the beads were resuspended with 150 μL freshly prepared 5% β-mercaptoethanol and incubated for 15 minutes in the dark. The beads were captured using a magnetic rack, and the supernatant was collected. This step was repeated with another 150 μL of warm 5% β-mercaptoethanol, and the 2 supernatants were combined (total 300 μL). Nascent RNA was precipitated using 2-propanol following a standard precipitation protocol. The extracted RNA was dissolved in 15 μL nuclease-free water and treated with DNase (iScript, Bio-Rad) to eliminate genomic DNA. cDNA synthesis was performed using the iScript kit (Bio-Rad), and qPCR was conducted using the primers listed in Supplemental Table 5.

### Bioinformatics and statistics.

For mRNA-seq, paired-end high-quality reads were mapped to the mouse genome mm10 (ENSEMBL version 86, GRCm38.p6) using STAR (51). Read counts for reads overlapping genomic regions were quantified using FeatureCounts (52), and analyzed using the DESeq2 R package for differential gene expression analysis (53). For mRNA-seq, paired-end high-quality reads were mapped to the mouse genome mm10 (ENSEMBL version 86, GRCm38.p6) using STAR. Read counts for reads overlapping genomic regions were quantified using FeatureCounts, and analyzed using the DESeq2 R package for differential gene expression analysis.

m^6^A-RIP-seq datasets were similarly preprocessed by aligning high-quality reads to the mouse genome mm10. To minimize the rate of false positives, only uniquely mapped reads were selected by SAMtools for downstream analyses. m^6^A peaks enriched in IP samples compared with corresponding input controls were identified using MACS2 (54). To identify m^6^A with high confidence, peaks were intersected in a pairwise fashion among 2 replicates using BEDTools (55). Fold changes for m^6^A peaks were obtained from MACS2 outputs. Consensus m^6^A peaks were mapped to CDSs, 5′ UTRs, 3′ UTRs, start codons, stop codons, and noncoding RNAs using intersectBed from BEDTools, according to RefSeq gene annotations (ENSEMBL version 86). The m^6^A consensus DRACH motif was identified via de novo motif search using HOMER (56), with m^6^A peaks as the target sequences and control peaks used as background. A Fisher’s exact test was used to determine whether m^6^A peaks were significantly altered between conditions (*P* < 0.05) with a log_2_(fold change) of greater than 1. GO analyses were performed on genes showing significantly differential m^6^A peaks. Biological process enrichment analyses were performed for the genes with highly increased/decreased m^6^A peaks using clusterProfiler (57).

### Statistics.

Unless otherwise specified, all statistical analyses were performed using GraphPad Prism software. Statistical significance was determined between 2 groups through paired or unpaired 2-tailed Student’s *t* test analysis when appropriate. For multiple groups, 1-way or 2-way ANOVA with Tukey’s or Šidák’s multiple-comparison test was used when appropriate to determine statistical significance. Two-way ANOVA for repeated measures was used for continuous measurements over time. All error bars shown represent SD, from at least 3 independent experiments. *P* values of less than 0.05 were considered statistically significant.

### Study approval.

Human samples were approved by the IEC for Clinical Research and Animal Trials of the First Affiliated Hospital of Sun Yat-Sen University (approval number 2021-668). Procedures for collecting human samples were approved by the Sydney Local Health District Human Ethics Committee (X20-0183). All patients provided written consent prior to participation. This study confirms with principles outlined in the Declaration of Helsinki (46). All animal experiments were approved by the Garvan Institute/St. Vincent’s Hospital Animal Ethics Committee (no. 20/04) and performed in accordance with NIH *Guide for the Care and Use of Laboratory Animals* (National Academies Press, 2011).

### Data availability.

Values for all graphs are available in the Supporting Data Values file in the supplemental material. Sequencing data have been deposited in the NCBI GEO repository with accession numbers GSE291558 and GSE291560. Additional data can be obtained from the corresponding authors upon reasonable request.

## Author contributions

GC and BSLL are co–first authors and contributed equally; the order of their names was based on duration of their involvement in the project. JJLW and RL conceived and designed the research. GC, BSLL, IDG, MM, and RL performed experiments and analyzed data. JT generated viruses. NP and QL prepared cells for mRNA-seq and m^6^A-RIP-seq. RS and JJLW performed bioinformatics analysis. DAR, MW, MMK, and JY provided clinical samples. GC, BSLL, JJLW, and RL wrote the manuscript, which was edited by MK and JY.

## Supplementary Material

Supplemental data

Unedited blot and gel images

Supporting data values

## Version 1 04/23/2025

In-Press Preview

## Version 2 05/22/2025

Electronic publication
