# Supplementary material for: METTL14 promotes intimal hyperplasia through m6A-mediated control of vascular smooth muscle dedifferentiation genes
Source: JCI Insight. 2025 Apr 17;10(10):e184444. doi: 10.1172/jci.insight.184444 (PMC12128973; doi:10.1172/jci.insight.184444)
Supplement: Supplemental data [file jciinsight-10-184444-s149.pdf]

SUPPLEMENTARY FIGURES

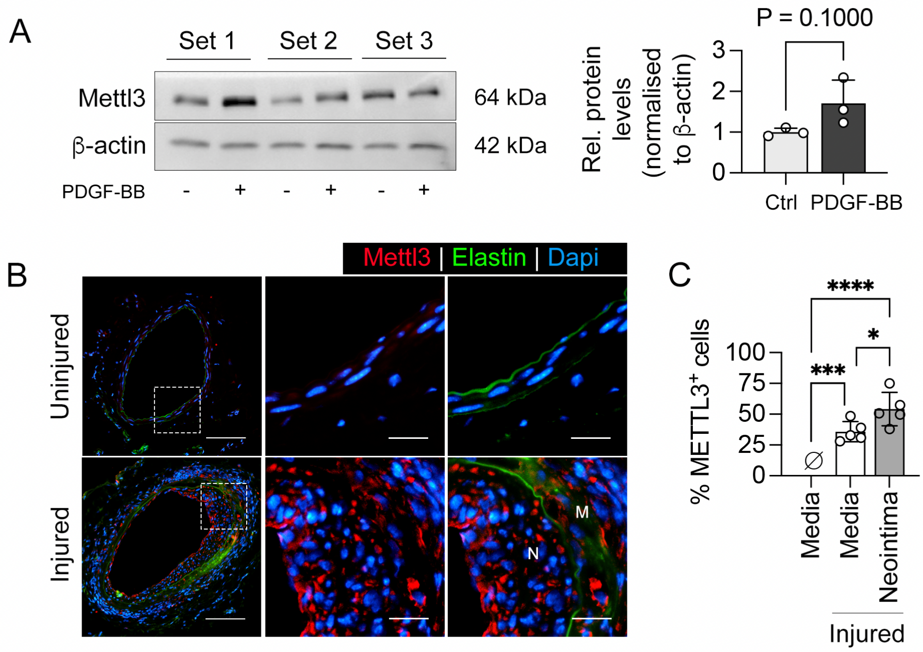

Supplemental Figure 1. Mettl3 expression is increased in injury-induced dedifferentiation of vascular smooth muscle cells. (**A**) Representative Western blot of Mettl3 in murine VSMCs treated without and with PDGF-BB for 72 h. *n* = 3 biologically independent repeats. Quantification shown on the right. (**B**) Representative immunofluorescent Mettl3 stained images of murine femoral arteries 21 days following femoral wire injury. Mettl3, red; elastin, green; Dapi, blue. Scale bar, 100 µm. (**C**) Quantification of Mettl3-positive nuclei in the media of uninjured femoral artery, media and neointima of injured femoral artery. *n* = 5 biologically independent samples. **P*<0.05, ****P*<0.005, *****P*<0.001; paired Student's *t*-test **(A)**; one-way ANOVA with Tukey's multiple comparisons test **(C)**. Error bars represent mean ± SD.

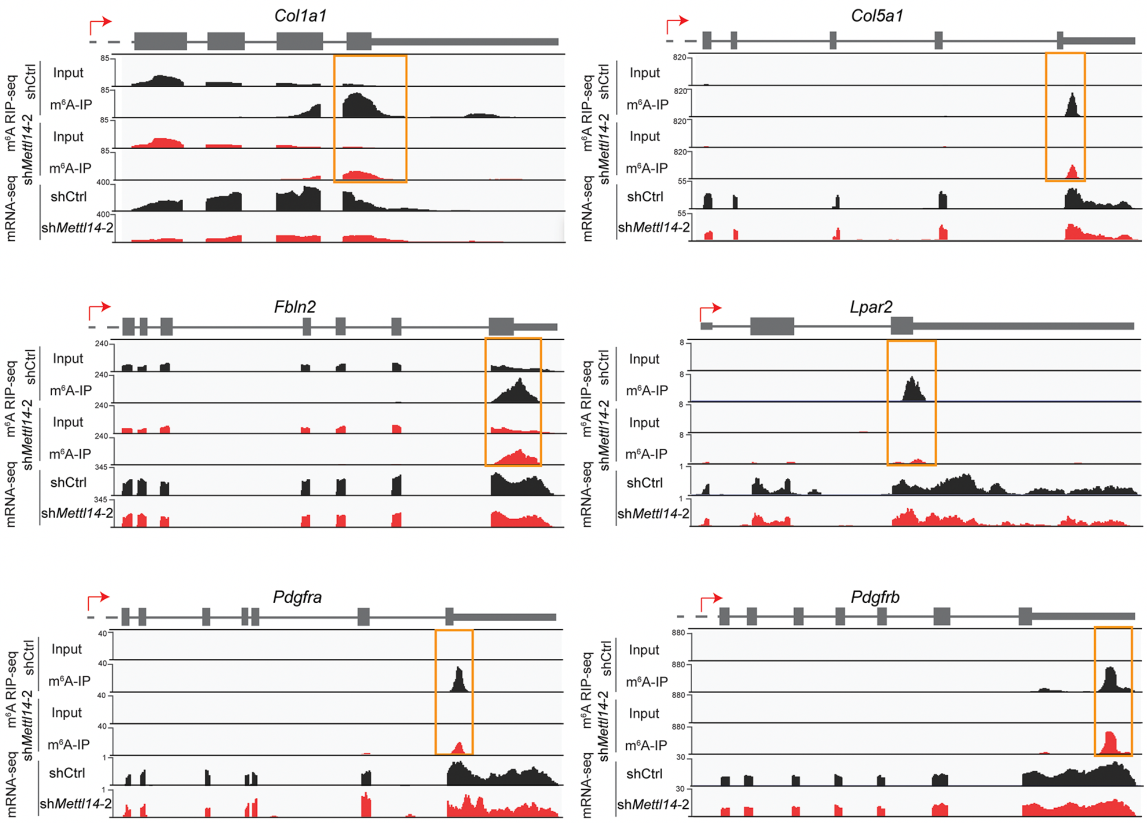

Supplemental Figure 2. Downregulation of m6A-modified genes involved in VSMC plasticity. Integrative Genome Viewer plots displaying m^{6}A peaks on *Col1a1*, *Col5a1*, *Fbln2*, *Lpar2*, *Pdgfra* and *Pdgfrb* mRNAs and the corresponding mRNA expression data in sh*Mettl14*-2 treated primary mouse VSMCs (red) and control (black). The m^{6}A peaks near stop codons of genes are indicated in the orange boxes.

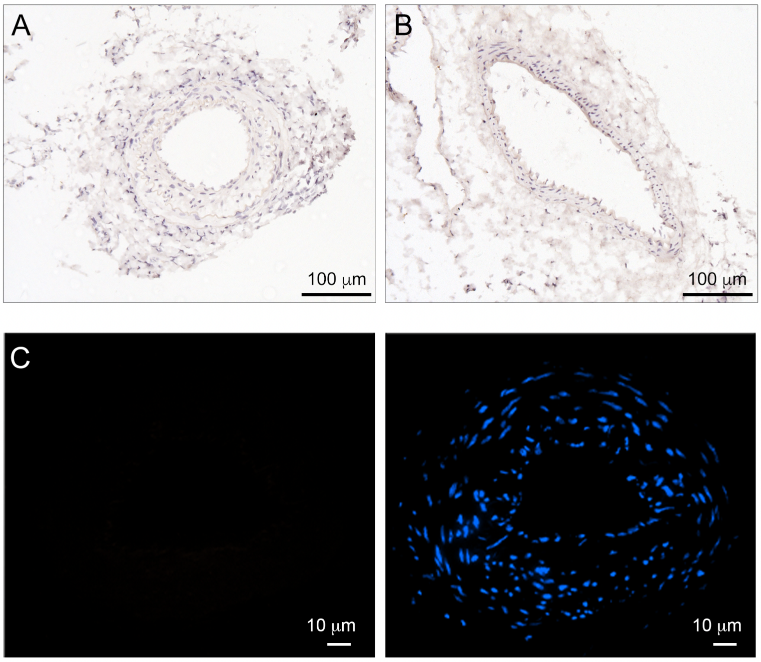

Supplemental Figure 3. Secondary antibody only control sections. Representative images of sections stained with **(A)** anti-mouse or **(B)** anti-rabbit HP conjugated secondary antibodies alone. Dapi counterstained with hematoxylin. **(C)** Representative image of sections stained with goat anti-rabbit IgG Alexa Fluor 488 secondary antibody only. Nuclear stained with Dapi.

Supplemental Table 1: Genes with significantly different m6A peaks between Mettl14 knckdown VSMCs and control

gene_name	peak coordinate	log2FoldChange	pvalue
Zcchc2	1:105991309-105991509	-1.079836834	0.04450
Zcchc2	1:106030880-106031221	0.734970429	0.04397
Sulf1	1:12692810-12693010	1.404146362	0.02915
Rab7b	1:131712256-131712553	0.901656518	0.02695
Abl2	1:156641592-156641851	-0.69199324	0.03849
Ildr2	1:166311949-166312149	1.82690805	0.02395
Gm7694	1:170301307-170301507	1.545151542	0.04893
Ndufs2	1:171247368-171247583	1.315055895	0.01136
Gulp1	1:44729270-44729470	1.386517508	0.02069
Mettl21a	1:64607685-64607885	0.858602427	0.03148
Tns1	1:73914034-73914538	-0.73867525	0.00368
Tuba4a	1:75216283-75216505	-0.585966585	0.04224
Per2	1:91442281-91442481	-1.556242403	0.00044
Mterf4	1:93301538-93301779	0.896475457	0.00966
Tmtc2	10:105413272-105413472	0.89327575	0.02773
Atxn7l3b	10:112927440-112927666	0.734164113	0.01155
Frs2	10:117074529-117074745	-0.613304437	0.03581
Plagl1	10:13128752-13128952	-1.130596229	0.01564
Ctgf	10:24597525-24597751	-0.616284328	0.03472
Mfsd4b4	10:39891555-39891755	1.268875369	0.01766
Mcm9	10:53537348-53537548	1.707765229	0.01204
Hk1	10:62269366-62269598	-0.681403588	0.01936
Trappc10	10:78189410-78189610	2.395777154	0.00177
2610008E11Rik	10:79067685-79067909	0.827965656	0.00598
Mbd3	10:80392739-80392939	-0.822076513	0.04331
Mob3a	10:80691253-80691453	-0.88986562	0.04079
Zbtb7a	10:81144835-81145035	-0.718484347	0.04634
Nuak1	10:84371240-84371468	-0.645558973	0.04642
Nuak1	10:84373894-84374119	-0.597019744	0.03999
Uhrf1bp1l	10:89813493-89813693	1.032148516	0.01103
Kat2a	11:100704875-100705128	0.989704683	0.01089
Itgb3	11:104643619-104643819	-0.986396781	0.03942
Slc16a6	11:109455576-109455776	1.768951283	0.00174
Cant1	11:118407632-118408053	-0.653761402	0.03915
C1qtnf1	11:118448247-118448480	0.634485621	0.04665
Gm11747	11:118448247-118448480	0.634485621	0.04665
Hexdc	11:121206506-121206706	0.845574107	0.04708
Sertad2	11:20647973-20648173	-1.018253662	0.01468
Morc2a	11:3689633-3689918	0.947599259	0.02955
Lif	11:4271690-4271977	-1.186955642	0.00538
Kremen1	11:5192274-5192474	-1.625227309	0.01214
Gm26551	11:52167787-52167987	-1.220341703	0.04946
Aff4	11:53415398-53415637	-0.91478578	0.03137
Slc36a1	11:55233627-55233859	-1.021009062	0.01978
2610507I01Rik	11:59199811-59200161	0.686642347	0.01941
Rai1	11:60185462-60185690	-1.092808629	0.00803
Mmgt2	11:62664952-62665152	0.987521299	0.02639
Borcs6	11:69060109-69060309	-1.094514061	0.02178
9330160F10Rik	11:69060109-69060309	-1.094514061	0.02178
Nlgn2	11:69825215-69825459	-1.073879342	0.03899
Derl2	11:71010043-71010346	-0.785563577	0.01983
Cyb5d2	11:72778119-72778353	-1.334496149	0.00637
Shpk	11:73223943-73224143	1.17756116	0.02694
Rnf135	11:80198718-80198965	-0.865152039	0.04404
Ptrh2	11:86689618-86689867	0.714389054	0.01870
Col1a1	11:94951491-94951691	-1.232322499	0.00038
Nfe2l1	11:96818332-96818532	-1.858983298	8.1356e-09
Nfe2l1	11:96820144-96820344	-0.97073669	0.00347
Nfe2l1	11:96827365-96827565	-1.157681795	0.00101

Socs7	11:97362843-97363043	-1.599207247	0.00980
Otub2	12:103404415-103404628	1.020977476	0.04227
Bag5	12:111710074-111710293	0.721851949	0.04075
Kif26a	12:112176174-112176417	1.538218509	0.00552
Trib2	12:15793835-15794075	0.677732499	0.04480
Fam110c	12:31074462-31074662	-1.336925754	0.03176
Snx13	12:35144172-35144383	0.917921633	0.03345
BC068281	12:4850884-4851084	1.152364572	0.03596
Six4	12:73103644-73103888	1.088293124	0.01474
Plekhg3	12:76576027-76576227	-0.923473756	0.02855
Ylpm1	12:85029405-85029617	0.614020945	0.04683
Irf2bpl	12:86881676-86882024	-0.597104537	0.04163
Ndufs4	13:114361318-114361518	1.1826716	0.04027
Yae1d1	13:17989418-17989618	-1.119475406	0.01321
Tubb2a	13:34074455-34074701	-0.734524881	0.03376
Hnrnpa0	13:58127482-58127682	-1.006013605	0.00447
Cdc14b	13:64196351-64196551	1.472219069	0.04113
Tert	13:73628386-73628586	1.143513976	0.03895
Zfp72	13:74371593-74371793	-1.608530868	0.00726
Fbxl3	14:103083140-103083340	-0.728933051	0.04429
Samd8	14:21775145-21775444	-0.898496491	0.03357
Btd	14:31667157-31667434	0.97499686	0.00637
9930012K11Rik	14:70154569-70154769	2.176813845	0.00274
Gm9174	14:70360315-70360548	-1.055104353	0.02327
Flnb	14:7951317-7951517	-1.084391351	0.00015
Elf1	14:79580484-79580684	-1.104028231	0.01629
4930556M19Rik	15:10721257-10721457	1.882932346	0.03308
Basp1	15:25364188-25364513	-0.880921973	0.00700
Basp1	15:25364670-25364905	-1.050987271	7.1397e-05
Myo10	15:25780889-25781107	-0.668019599	0.04302
Ghr	15:3518855-3519072	0.677742241	0.02109
Ncald	15:37397119-37397319	2.030072801	0.00572
Trmt12	15:58873129-58873379	1.07046087	0.02000
Jrk	15:74706560-74706760	1.819792989	0.00318
Plec	15:76176249-76176456	-0.657522746	0.04431
Plec	15:76177188-76177636	-0.586600286	0.01560
Tnrc6b	15:80879187-80879408	0.713813572	0.03713
Fam118a	15:85061688-85061906	-0.991413192	0.01528
Gxylt1	15:93244623-93245064	0.619523656	0.03011
Lsg1	16:30571326-30571526	-0.972020156	0.03035
Nrros	16:32143490-32143690	2.102914873	0.00063
Slx4	16:3979663-3979868	-0.793753543	0.04008
Slx4	16:3986924-3987155	-0.964305132	0.01231
Plcxd2	16:45963776-45964012	-0.943353059	0.02707
Gm15638	16:45963776-45964012	-0.943353059	0.02707
Nxpe3	16:55844086-55844286	1.916135495	0.01692
Col8a1	16:57628101-57628313	-0.715104983	0.03982
Robo1	16:72663975-72664175	1.057063594	0.04340
Chd1	17:16973403-16973700	0.775508065	0.03338
BC002059	17:16973403-16973700	0.775508065	0.03338
Zfp944	17:22339686-22339886	1.241270625	0.01887
Axin1	17:26143299-26143499	0.918500248	0.03564
Ggnbp1	17:26975989-26976189	1.417826948	0.01053
Zbtb9	17:26975989-26976189	1.417826948	0.01053
Btbd9	17:30217977-30218202	-1.061571794	0.01809
Gm26549	17:32285160-32285383	1.006507484	0.00852
Tjap1	17:46258593-46258802	-0.639048587	0.04919
Arid1b	17:4995695-4995900	-0.943775964	0.03047
Ubxn6	17:56067355-56067555	-0.846658994	0.04877
Chaf1a	17:56067355-56067555	-0.846658994	0.04877
Synj2	17:6043733-6043957	0.821107569	0.04655
Serac1	17:6043733-6043957	0.821107569	0.04655
Tgif1	17:70844810-70845054	-0.689101219	0.04363

Fam53c	18:34768420-34768643	-1.04052783	0.00827
Gm37013	18:37696612-37696853	0.838807277	0.04492
Pcdhga3	18:37696612-37696853	0.838807277	0.04492
Pcdhgb1	18:37696612-37696853	0.838807277	0.04492
Pcdhga4	18:37696612-37696853	0.838807277	0.04492
Gm42416	18:37696612-37696853	0.838807277	0.04492
Pcdhga2	18:37696612-37696853	0.838807277	0.04492
Gm37388	18:37696612-37696853	0.838807277	0.04492
Pcdhgb2	18:37696612-37696853	0.838807277	0.04492
Pcdhga5	18:37696612-37696853	0.838807277	0.04492
Pcdhga1	18:37696612-37696853	0.838807277	0.04492
Gm37013	18:37767848-37768048	1.145617238	0.04869
Gm26672	18:37767848-37768048	1.145617238	0.04869
Pcdhga12	18:37767848-37768048	1.145617238	0.04869
Pcdhga9	18:37767848-37768048	1.145617238	0.04869
Pcdhga5	18:37767848-37768048	1.145617238	0.04869
Pcdhgb7	18:37767848-37768048	1.145617238	0.04869
Pcdhga7	18:37767848-37768048	1.145617238	0.04869
Pcdhga2	18:37767848-37768048	1.145617238	0.04869
Gm42416	18:37767848-37768048	1.145617238	0.04869
Gm37388	18:37767848-37768048	1.145617238	0.04869
Pcdhga3	18:37767848-37768048	1.145617238	0.04869
Pcdhga4	18:37767848-37768048	1.145617238	0.04869
Pcdhga1	18:37767848-37768048	1.145617238	0.04869
Pcdhga6	18:37767848-37768048	1.145617238	0.04869
Pcdhgb2	18:37767848-37768048	1.145617238	0.04869
Pcdhgb5	18:37767848-37768048	1.145617238	0.04869
Pcdhga8	18:37767848-37768048	1.145617238	0.04869
Pcdhga10	18:37767848-37768048	1.145617238	0.04869
Pcdhgb1	18:37767848-37768048	1.145617238	0.04869
Pcdhgb4	18:37767848-37768048	1.145617238	0.04869
Pcdhga11	18:37767848-37768048	1.145617238	0.04869
Pcdhgb8	18:37767848-37768048	1.145617238	0.04869
Pcdhgb6	18:37767848-37768048	1.145617238	0.04869
Sema6a	18:47248662-47248862	1.184441439	0.04410
Sncaip	18:52915304-52915597	0.976793597	0.04007
Zfp608	18:54988281-54988481	1.751803494	0.00530
Zeb1	18:5727730-5727930	1.953561563	0.00276
Slc12a2	18:57879153-57879353	-1.104538019	0.00716
Ndst1	18:60713250-60713514	-1.149647098	0.00058
Adrb2	18:62178488-62178703	0.780753625	0.03735
Zadh2	18:84095049-84095354	-0.798411516	0.01299
Osbp	19:11991859-11992101	-0.735717885	0.01491
Jak2	19:29311707-29311907	-1.788156075	0.00623
Ankrd1	19:36112113-36112320	-0.942903856	0.00813
Marveld1	19:42151363-42151563	-1.116340564	0.04917
R3hcc1l	19:42590914-42591120	1.183109702	0.00224
Wbp1l	19:46655932-46656132	-1.125276458	0.01452
Tmem151a	19:5074581-5074781	1.133604102	0.02534
Ccdc85b	19:5457015-5457512	-0.90676838	0.00364
Rtn3	19:7456636-7456836	-1.044206951	0.04769
Prrg4	2:104832356-104832556	1.588780067	0.04761
Rpusd2	2:119038425-119038628	0.865712878	0.03516
Vps18	2:119293660-119293881	-0.649800126	0.04541
1700020I14Rik	2:119599677-119599893	-0.624091807	0.02282
Stard9	2:120696532-120696732	-1.859208123	0.00331
Zscan29	2:121163988-121164188	-1.09546971	0.04209
Gm14005	2:128252825-128253025	-2.844380522	0.03416
Pced1a	2:130423787-130423987	0.854850826	0.03595
A730017L22Rik	2:130874386-130874586	0.865838951	0.02072
4930402H24Rik	2:130874386-130874586	0.865838951	0.02072
Hacd1	2:14033497-14033697	1.029074891	0.03437
Rrbp1	2:143988756-143988959	-0.793708961	0.03033

Rrbp1	2:143989459-143989659	-1.117672889	0.00179
Gzf1	2:148683717-148683917	1.376768303	0.00833
Csnk2a1	2:152267519-152267719	1.116729736	0.02668
Sox12	2:152396850-152397050	1.238424076	0.04284
Mcts2	2:152687359-152687559	-1.043552029	0.03241
H13	2:152687359-152687559	-1.043552029	0.03241
E2f1	2:154560642-154560842	-1.631586661	0.00184
Ncoa6	2:155405643-155405883	-0.70324043	0.02570
Dlgap4	2:156762855-156763106	-0.89769615	0.04719
Tgif2	2:156853638-156853894	1.362491823	0.02749
5430405H02Rik	2:156853638-156853894	1.362491823	0.02749
Tti1	2:158007329-158007529	1.149900152	0.01865
Fam83d	2:158785394-158785629	-1.231419893	0.00993
Zmynd8	2:165807801-165808043	0.944263625	0.01680
Zmynd8	2:165812415-165812757	0.626503734	0.01900
Zbtb46	2:181391200-181391400	-1.62950113	0.00247
Etl4	2:20806429-20806657	0.751960677	0.03466
Tor4a	2:25195576-25195821	-0.959285576	0.01287
Ntng2	2:29227676-29227904	1.120219903	0.01690
Cercam	2:29882436-29882636	-1.263922534	0.01068
Dolpp1	2:30399182-30399382	-1.079062041	0.01591
Dhrs9	2:69401814-69402014	2.339309599	7.8856e-05
Tnks1bp1	2:85052264-85052486	-0.941814111	0.00491
Trim45	3:100925330-100925573	1.05866057	0.01432
Ngf	3:102520264-102520464	-0.80915702	0.00265
Ngf	3:102520494-102520762	-0.650073793	0.01272
Magi3	3:104014869-104015097	0.67080092	0.03313
Phtf1	3:104014869-104015097	0.67080092	0.03313
Dph5	3:115906696-115906896	-1.628041675	0.03251
Lrrc39	3:116574899-116575107	1.739255265	0.03359
Rwdd3	3:121158786-121158998	1.105899918	0.00489
Alpk1	3:127679806-127680048	0.963635893	0.04067
Sgms2	3:131322929-131323152	-0.64082985	0.02481
Tet2	3:133466905-133467134	0.903115551	0.03726
Gbp7	3:142547952-142548152	-1.513216994	0.00729
Gm43802	3:142547952-142548152	-1.513216994	0.00729
Pigk	3:152788741-152788941	-0.739542157	0.03659
Pld1	3:28131759-28131984	0.590077837	0.04009
Mecom	3:29979640-29979880	0.984546054	0.00968
D3Ertd254e	3:36165736-36165981	1.180871891	0.00149
Nudt6	3:37404720-37404920	-0.959476115	0.04402
Fgf2	3:37404720-37404920	-0.959476115	0.04402
Gm43439	3:37404720-37404920	-0.959476115	0.04402
Ankrd50	3:38451687-38451888	-0.666552217	0.03748
Il6ra	3:89870104-89870343	0.927615164	0.03839
Sprr2a2	3:92242018-92242218	6.364830146	2.1537e-06
Zbtb10	3:9251770-9251970	-1.240575829	0.00725
Zfp687	3:95012031-95012280	1.016447936	0.00761
Hist2h2be	3:96221309-96221509	2.411843185	2.9735e-06
1110037F02Rik	4:11519132-11519332	0.946269144	0.04372
Hivep3	4:120133871-120134077	1.387696096	0.00045
Macf1	4:123434717-123434956	-0.654670674	0.02690
Zmym1	4:127048934-127049134	2.424066499	0.00246
Gm12940	4:127048934-127049134	2.424066499	0.00246
Bsdc1	4:129487512-129487734	0.729341049	0.03589
Ahdc1	4:133066011-133066221	-1.138168062	0.00727
Fam46b	4:133487011-133487224	0.959845383	0.02975
Arid1a	4:133685163-133685363	-0.691195168	0.04421
Arid1a	4:133752944-133753144	-1.139132919	0.01012
Cep85	4:134162744-134162944	-1.935992764	0.02333
Runx1t1	4:13890235-13890435	2.067133577	0.00499
Tmem51	4:142031593-142031820	-0.966445223	0.02753
Fndc10	4:155695357-155695557	1.3096307	0.02902

Isg15	4:156199615-156199815	-1.257481304	0.02089
Epha7	4:28821066-28821394	0.82104162	0.02543
B4galt1	4:40806825-40807025	-1.154879625	0.01319
Fam214b	4:43035895-43036095	-1.275167041	0.00511
Tbc1d2	4:46604894-46605094	1.2585897	0.02168
Impad1	4:4767608-4767848	-0.598652988	0.01869
Zfp189	4:49529080-49529280	-1.105380722	0.03102
Zfp462	4:55010294-55010506	0.699657289	0.02837
Zfp462	4:55011468-55011677	0.842898304	0.04176
Klf4	4:55530035-55530235	-1.068176592	0.02804
Slc31a2	4:62297218-62297418	-1.177986107	0.04555
Tlr4	4:66839544-66839804	-1.069820023	0.01993
Brinp1	4:68761897-68762157	0.995036883	0.02157
Brinp1	4:68762400-68762600	1.4761703	0.00342
C630043F03Rik	4:72201325-72201525	1.229980316	0.02858
A830010M20Rik	5:107507466-107507666	1.269911207	0.04065
Gm42669	5:107507466-107507666	1.269911207	0.04065
Coro1c	5:113875200-113875400	1.795797842	0.01375
Gm15800	5:121220365-121220565	-1.374047106	0.03475
Sfswap	5:129501252-129501452	-0.937936962	0.02092
Clip2	5:134490517-134490728	-0.958910914	0.01335
Tbl2	5:135159353-135159586	0.936068421	0.04697
Serpine1	5:137062891-137063118	-0.907751792	0.00391
Ephb4	5:137373614-137373849	-1.087351184	0.02553
Zkscan1	5:138104383-138104612	0.785926213	0.04432
Zfand2a	5:139473084-139473323	-0.777676233	0.03527
Chst12	5:140523841-140524107	-0.979998087	0.00072
Chst12	5:140524680-140524972	-0.615348017	0.02165
Rnf6	5:146211690-146211909	-0.914097448	0.03451
Kmt2c	5:25310415-25310615	1.001099047	0.04355
Emilin1	5:30921031-30921231	-0.931922708	0.00805
Preb	5:30951742-30951942	1.534827005	0.03025
Abhd1	5:30951742-30951942	1.534827005	0.03025
Ppm1g	5:31206106-31206306	-0.720453275	0.03120
Bloc1s4	5:36747797-36748023	-0.891829851	0.00546
Bloc1s4	5:36748184-36748414	-1.058161316	0.00491
Fzd1	5:4754169-4754369	-0.921067879	0.04839
Slit2	5:47984164-47984378	0.775316417	0.03030
Slit2	5:48304464-48304664	0.807130078	0.03167
Stim2	5:54118917-54119117	0.828734388	0.03832
Stim2	5:54119125-54119325	1.256919696	0.00329
G3bp2	5:92054500-92054700	-1.363728477	0.00430
Gm26582	5:92054500-92054700	-1.363728477	0.00430
Srgap3	6:112719048-112719376	0.961040143	0.04109
Tatdn2	6:113702260-113702460	-0.771927527	0.03925
Csgalnact2	6:118129079-118129279	-0.853126868	0.04987
Wnk1	6:119962665-119962865	-2.204236079	0.00989
Wnk1	6:119963189-119963389	-1.822060078	0.00823
Mical3	6:120931886-120932143	0.801817088	0.03587
Ptms	6:124914651-124914851	-0.652551702	0.04677
Vamp1	6:125240577-125240841	-0.97032399	0.02189
9330102E08Rik	6:128171905-128172145	0.947492964	0.03954
Gcc1	6:28418249-28418449	-1.035708352	0.01907
Mrpl19	6:81961928-81962128	1.231864716	0.00909
Sfxn5	6:85214426-85214626	2.617998241	0.00554
Fbln2	6:91234076-91234315	-0.947954427	0.00111
Fbln2	6:91250744-91250975	-0.760732139	0.02111
Fbln2	6:91252338-91252564	-1.471700118	0.00256
Fbln2	6:91254346-91254546	-1.854753907	0.00181
Lipt2	7:100160396-100160596	1.487838289	0.04271
Fam160a2	7:105389580-105389780	1.533215971	0.00616
Dchs1	7:105758730-105758930	-1.145655501	0.04273
Sox6	7:115503411-115503721	1.615757765	0.03364

Itpripl2	7:118489642-118489866	-0.898046065	0.00159
Itpripl2	7:118490807-118491036	-0.627443731	0.02121
Ccp110	7:118722643-118722843	1.245662263	0.00327
Knop1	7:118844464-118844664	0.996922229	0.03975
Tnrc6a	7:123171467-123171667	0.94324737	0.00540
Zfp606	7:12493597-12493797	1.119890966	0.04995
AC139131.1	7:12493597-12493797	1.119890966	0.04995
Bola2	7:126695548-126695748	-1.295914942	0.03291
Slx1b	7:126695548-126695748	-1.295914942	0.03291
9130019O22Rik	7:127384189-127384418	1.174317436	0.01282
Prr14	7:127459768-127459987	0.62888502	0.03886
B130055M24Rik	7:127459768-127459987	0.62888502	0.03886
Setd1a	7:127785188-127785406	-0.830071154	0.03584
Phrf1	7:141258168-141258452	0.906348581	0.03355
Mrgpre	7:143780672-143780882	-1.199844676	0.00560
Ccdc8	7:16994234-16994434	1.062965941	0.02860
Sipa1l3	7:29400834-29401034	-0.936146802	0.04856
Prkcg	7:3302244-3302444	-2.682765383	8.9093e-07
Zfp976	7:42609787-42610001	1.199899931	0.02704
Zfp976	7:42610102-42610302	1.947710022	0.00226
Lmtk3	7:45798190-45798390	-2.236916134	0.02980
Asb7	7:66647790-66647990	0.855758512	0.04652
Ttc23	7:67734799-67735007	0.795381287	0.01916
Synm	7:67734799-67735007	0.795381287	0.01916
Acan	7:79099807-79100042	-0.771214666	0.03629
Zfp592	7:81041633-81041833	-0.83357511	0.03699
Gab2	7:97299071-97299271	-1.254074513	0.02639
Rnf169	7:99924346-99924546	1.231972984	0.01170
Rnf169	7:99925477-99925710	0.70532109	0.01649
Nob1	8:107412641-107412841	-1.87848444	0.00041
Zfp1	8:111669622-111669846	0.800623406	0.00685
Bcar1	8:111710613-111710813	-0.704926968	0.02219
Gm20388	8:122264886-122265086	-2.042059712	0.00103
Zfpm1	8:122336640-122336840	-2.650324582	0.00018
Gm20388	8:122336640-122336840	-2.650324582	0.00018
Exoc8	8:124896544-124896745	-0.804547597	0.02733
Erlin2	8:27036528-27036766	0.703453609	0.01571
Cdkn2aip	8:47711160-47711415	0.825158636	0.01929
Lpar2	8:69826523-69826723	-3.58673466	2.6215e-08
Crtc1	8:70386052-70386252	-1.293199659	0.01576
Gm11175	8:70699012-70699212	-0.986236699	0.00131
Jund	8:70699012-70699212	-0.986236699	0.00131
Prmt9	8:77552486-77552686	1.918384514	0.00953
Tmem184c	8:77596561-77596864	0.668059099	0.01552
Arglu1	8:8667220-8667420	0.791410887	0.02491
Rspry1	8:94628939-94629139	1.68233402	0.01934
Slco2a1	9:103087293-103087536	-0.790421851	0.01745
Ctdspl	9:119013735-119013935	1.843110087	0.01334
Keap1	9:21237433-21237633	-1.895594538	0.00300
Nlrx1	9:44252789-44252989	1.269440425	0.03850
Zc3h12c	9:52116138-52116343	0.717134028	0.03785
Zc3h12c	9:52116473-52116673	1.326915769	0.00627
Peak1	9:56283218-56283478	1.070695733	0.01265
Cspg4	9:56887789-56887989	-1.31928131	0.00249
Cspg4	9:56898772-56899001	-0.770429518	0.01308
Cspg4	9:56899548-56899748	-1.127461079	0.00721
Glce	9:62070162-62070408	-0.83436869	0.02695
Tpbg	9:85844838-85845043	0.883150306	0.02010
Ercc6l	X:102144994-102145194	-1.22447549	0.04932
Brwd3	X:108743354-108743583	0.661465345	0.02875
Iqsec2	X:152209384-152209584	-1.004980975	0.03979
Cybb	X:9484936-9485136	1.301393823	0.01962

Supplemental Table 2: The primary antibodies used in immunofluorescence staining

Antibody	Isotype	Source	Catalogue number	Conjugate	Working dilution
METTL14	Rabbit	Abclonal	A8530	-	1:200
METTL3	Rabbit	Abclonal	A8370	-	1:200
SERPINE1	Rabbit	Abcam	ab66705	-	1:200
KLF4	Rabbit	Abclonal	A1363	-	1:2000
p-STAT1	Rabbit	Cell signalling	91675	-	1:100
STAT1	Rabbit	Cell signalling	14994	-	1:100
ACTA2	Mouse	Sigma	C6198	Cy3	1:5000
Goat anti-rabbit IgG	Rabbit	Invitrogen	A32733	Alexa fluor plus 647	1:500
Goat anti-rabbit IgG	Rabbit	Invitrogen	A32740	Alexa fluor plus 594	1:500

Supplemental Table 3: qRT-PCR primer sequences

Primer name	Forward	Reverse
Ythdc1	TTCAGGAGTTCGCCGAGATG	TCCCGGGTAAGGAGGATTCC
Ythdf1	GGAGGTGGTGCGTAAGGAAA	GACGACACTGGAGCTGACTC
Ythdf2	GGCAAGGCCGAATAATGCAT	CCTCCAGTAGACCAAGCAGC
Mettl3	GAGGTTCGTTCCACCAGTCA	A TCCAGTTGGGCTGCACA TT
Mettl14	CATCAGGGATGTAGGTTTAGCTG	TGGGAGGAGTGTTTGACTTAGC
Virma	GCCGCTTAGTTCTACGGGTT	CTTTCCTCTCGAGCCTCCAC
Wtap	AAAGCAGCAACAGCAGGAGT	ACTGGATTTGAGTGGTGCAC
Fto	GCATGGCGCTGAAATACCC	TTGCCTTGGATCCTCACCAC
Alkbh5	TGTGCTCAGTGGGTATGCTG	TCCAATCGCGGTGCATCTAA
Mmp1	TTGGCTTAGAGGTGACTGGC	TTCACCCACATCAGGCACTC
Mmp2	CAAGGATGGACTCCTGGCACAT	TACTCGCCATCAGCGTTCCCAT
Mmp3	CTCTGGAACCTGAGACATCACC	AGGAGTCCTGAGAGATTTGCGC
Mmp7	AGGTGTGGAGTGCCAGATGTTG	CCACTACGATCCGAGGTAAGTC
Mmp9	GCTGACTACGATAAGGACGGCA	TAGTGGTGCAGGCAGAGTAGGA
Mmp10	TGCTGCCTATGAGGCTCACAAC	GGAGGAAAACCGAGAGTGTGGA
Mmp12	CACACTTCCCAGGAATCAAGCC	TTTGGTGACACGACGGAACAGG
18S	CCGCAGCTAGGAATAATGGA	CCCTCTTAATCATGGCCTCA

Supplemental Table 4: The primary antibodies used in Western blots

Antibody	Isotype	Source	Catalogue number	Conjugate	Working dilution
METTL14	Rabbit	Sigma	HPA038002	-	1:1000
METTL3	Rabbit	Abcam	Ab195352	-	1:1000
SERPINE1	Rabbit	Abcam	Ab66705	-	1:800
CALPONIN	Mouse	ThermoFisher	MA511620	-	1:2000
ACTA	Mouse	Abcam	ab7817	-	1:2000
TAGLN	Rabbit	Abcam	ab14106	-	1:2000
MYH11	Mouse	Sigma	M7786	-	1:1000
MYH10	Mouse	ThermoFisher	MA527767	-	1:1000
KLF4	Rabbit	ThermoFisher	PA1095	-	1:500
OPN	Rabbit	ThermoFisher	702184		1:1000
p-STAT1	Rabbit	Cell signalling	91675	-	1:1000
STAT1	Rabbit	Cell signalling	149945	-	1:1000
β-actin	Mouse	Abcam	ab49900	HRP	1:12000
Anti-mouse IgG	Mouse	Cell signalling	7076	HRP	1:5000
Anti-rabbit IgG	Rabbit	Cell signalling	7074	HRP	1:5000

Supplemental Table 5: qRT-PCR primer sequences for nascent RNA assay

Primer name	Forward	Reverse
mKLF4_4sU	CCCCTCTCTCTTCTTCGGAC	CAGTGTCTTCTCCCTTCCCG
mSerpine1_4sU	GCCAACAAGAGCCAATCACA	GACCACCTGCTGAAACACTT
mACTB_4sU	TCTTTGCAGCTCCTTCGTTG	ACGATGGAGGGGAATACAGC
