# Supplementary material for: METTL14 promotes intimal hyperplasia through m6A-mediated control of vascular smooth muscle dedifferentiation genes
Source: JCI Insight. 2025 Apr 17;10(10):e184444. doi: 10.1172/jci.insight.184444 (PMC12128973; doi:10.1172/jci.insight.184444)

Figure 1a

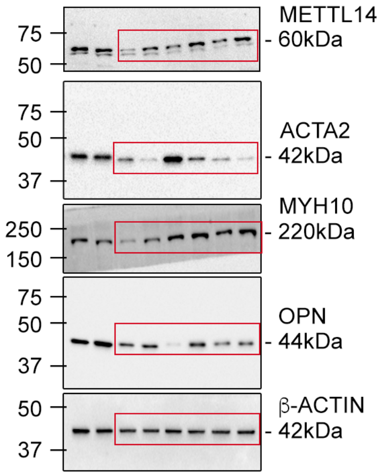

Figure 2a

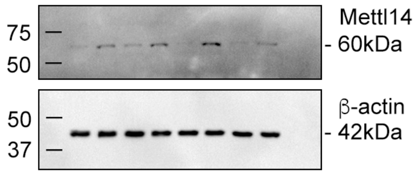

Figure 5a

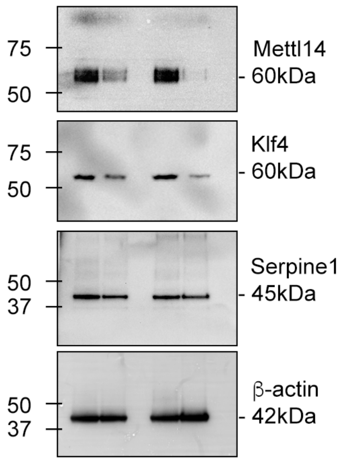

Figure 3a

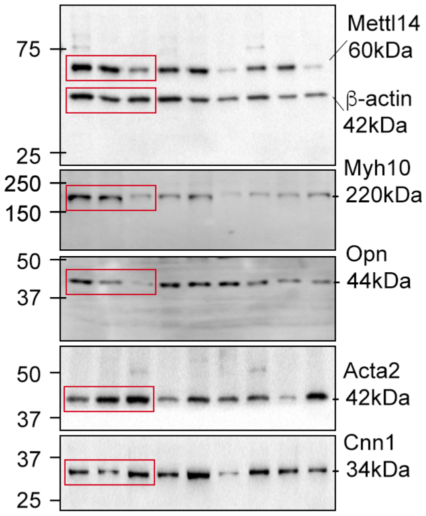

Figure 6a

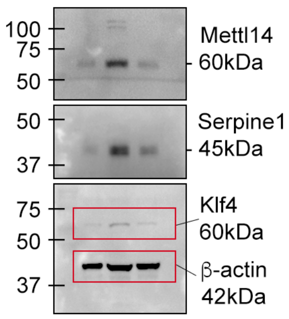

Figure 5i

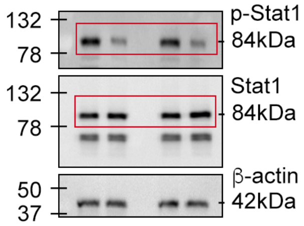

Figure 6c

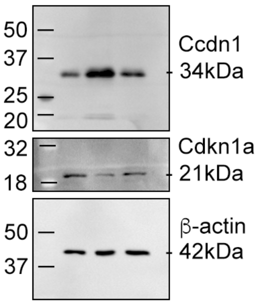

Figure 6e

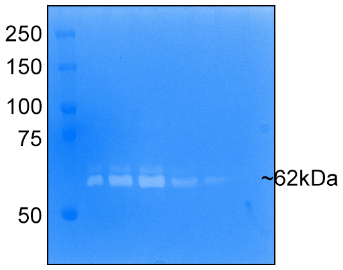

Figure 6f

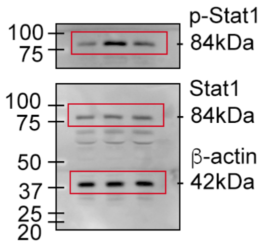

Figure 6g

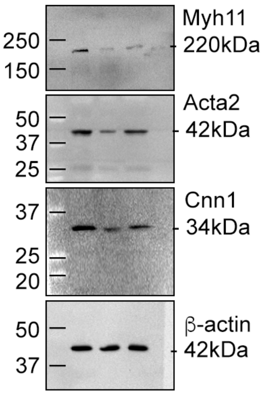

Supplemental figure 1a

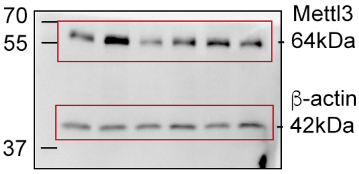

Supplement: Unedited blot and gel images [file jciinsight-10-184444-s150.pdf]
